# The Evolution of Azole Resistance in *Candida albicans* Sterol 14α-Demethylase (CYP51) through Incremental Amino Acid Substitutions

**DOI:** 10.1128/AAC.02586-18

**Published:** 2019-04-25

**Authors:** Andrew G. Warrilow, Andrew T. Nishimoto, Josie E. Parker, Claire L. Price, Stephanie A. Flowers, Diane E. Kelly, P. David Rogers, Steven L. Kelly

**Affiliations:** a Institute of Life Science, Swansea University Medical School, Swansea University, Swansea, Wales, United Kingdom; b Department of Clinical Pharmacy and Translational Science, College of Pharmacy, University of Tennessee Health Science Center, Memphis, Tennessee, USA; c University of Tennessee Center for Pediatric Experimental Therapeutics, Memphis, Tennessee, USA

**Keywords:** *Candida albicans* CYP51, azole, mutations

## Abstract

Recombinant Candida albicans CYP51 (CaCYP51) proteins containing 23 single and 5 double amino acid substitutions found in clinical strains and the wild-type enzyme were expressed in Escherichia coli and purified by Ni^2+^-nitrilotriacetic acid agarose chromatography. Catalytic tolerance to azole antifungals was assessed by determination of the concentration causing 50% enzyme inhibition (IC_50_) using CYP51 reconstitution assays. The greatest increase in the IC_50_ compared to that of the wild-type enzyme was observed with the five double substitutions Y132F+K143R (15.3-fold), Y132H+K143R (22.1-fold), Y132F+F145L (10.1-fold), G307S+G450E (13-fold), and D278N+G464S (3.3-fold). The single substitutions K143R, D278N, S279F, S405F, G448E, and G450E conferred at least 2-fold increases in the fluconazole IC_50_, and the Y132F, F145L, Y257H, Y447H, V456I, G464S, R467K, and I471T substitutions conferred increased residual CYP51 activity at high fluconazole concentrations. *In vitro* testing of select CaCYP51 mutations in C. albicans showed that the Y132F, Y132H, K143R, F145L, S405F, G448E, G450E, G464S, Y132F+K143R, Y132F+F145L, and D278N+G464S substitutions conferred at least a 2-fold increase in the fluconazole MIC. The catalytic tolerance of the purified proteins to voriconazole, itraconazole, and posaconazole was far lower and limited to increased residual activities at high triazole concentrations for certain mutations rather than large increases in IC_50_ values. Itraconazole was the most effective at inhibiting CaCYP51. However, when tested against CaCYP51 mutant strains, posaconazole seemed to be the most resistant to changes in MIC as a result of CYP51 mutation compared to itraconazole, voriconazole, or fluconazole.

## Abstract (precis)

Recombinant Candida albicans CYP51 (CaCYP51) proteins containing 23 single and 5 double amino acid substitutions found in clinical strains and the wild-type enzyme were expressed in Escherichia coli and purified by Ni^2+^-nitrilotriacetic acid agarose chromatography. Catalytic tolerance to azole antifungals was assessed by determination of the concentration causing 50% enzyme inhibition (IC_50_) using CYP51 reconstitution assays.

## INTRODUCTION

Candida albicans causes a wide range of clinical infections in humans, ranging from mucosal infections, such as oral candidiasis and thrush, to potentially life-threatening systemic candidemia and candidiasis. The incidence of fungal infections caused by C. albicans and non-*albicans Candida* species has steadily increased over the last 2 decades in part due to HIV-AIDS but also as a result of the growing number of patients who are immunodeficient through organ and bone marrow transplants and cancer treatments (1). *Candida* bloodstream infections cause significant mortality and morbidity, particularly among intensive care patients (2, 3). Predisposing factors for invasive candidiasis include cytotoxic and immunosuppressive therapies, HIV-AIDS, treatment with broad-spectrum antibiotics, diabetes, and *in situ* urinary tract and central venous catheters (4–6). Controlling invasive fungal infections among oncology, hematology, and intensive care patients is a growing concern (7).

Azole antifungals are relatively safe, have a high therapeutic index, and are easy to administer (often by mouth), leading to the adoption of triazole antifungals as the standard first-line therapy against fungal infections. However, prolonged treatment regimens in the clinic and the prophylactic use of azole drugs have led to an increasing incidence of azole-resistant *Candida* strains. Four molecular mechanisms have been demonstrated to confer azole resistance in *Candida* (8, 9). These include overexpression of efflux transporters (C. albicans Cdr1 [CaCdr1], CaCdr2, and CaMdr1) (10, 11), point mutations that alter the amino acid sequence of CYP51 (*ERG11*) (12–14), overexpression of CYP51, and alterations in the ergosterol biosynthetic pathway, such as mutations in *ERG3*. These resistance mechanisms are often combined in clinical isolates (15–20).

More than 140 different amino acid substitution mutations have been reported in the C. albicans CYP51 (CaCYP51) gene from clinical strains, although not all substitutions confer an azole resistance phenotype (13, 14, 21–23). The CYP51 genes of both azole-susceptible and azole-resistant clinical isolates may contain several amino acid substitutions. The majority of these mutations cluster into three hot spots, located within residues 105 to 165, 266 to 287, and 405 to 488 (21). Not all amino acid substitutions contribute equally to azole resistance, with K143R, S405F, G464S, R467K, and I471T being present only in CYP51 genes recovered from azole-resistant strains, whereas E266D, V437I, and V488I have been found in CYP51 genes from both azole-susceptible and azole-resistant strains, suggesting that the last three substitutions do not confer azole resistance (23). CYP51-mediated azole resistance has been investigated further through genetic manipulation by introducing CYP51 genes on plasmids into a model Saccharomyces cerevisiae system and then assessing azole sensitivity (14, 24, 25). In addition, several CaCYP51 constructs of the enzyme catalytic domain containing amino acid substitutions, including Y132H, F145L, I471T, and S279F, have been expressed in Escherichia coli, purified, and characterized (26–29).

In this study, we report the expression in E. coli, isolation, and purification of recombinant CaCYP51 proteins that were modified at the N terminus and His tagged at the C terminus and that had 1 of 23 single amino acid substitutions or 5 double amino acid substitutions. The effects of the amino acid substitutions were assessed in terms of catalytic turnover and sensitivity to inhibition by fluconazole, itraconazole, voriconazole, and posaconazole using CYP51 reconstitution assays and azole ligand binding studies. The effects of selected single and double amino acid substitutions in CaCYP51 proteins on susceptibility were further tested *in vitro* by introducing mutant alleles of the CYP51 gene into an azole-susceptible C. albicans strain. The aim of this study was to establish which CaCYP51 amino acid substitutions conferred biochemical azole tolerance to the CYP51 catalytic function and their impact on *in vitro* azole susceptibility.

## RESULTS

### Purification and enzyme catalysis of CaCYP51 proteins.

**FIG 1:**
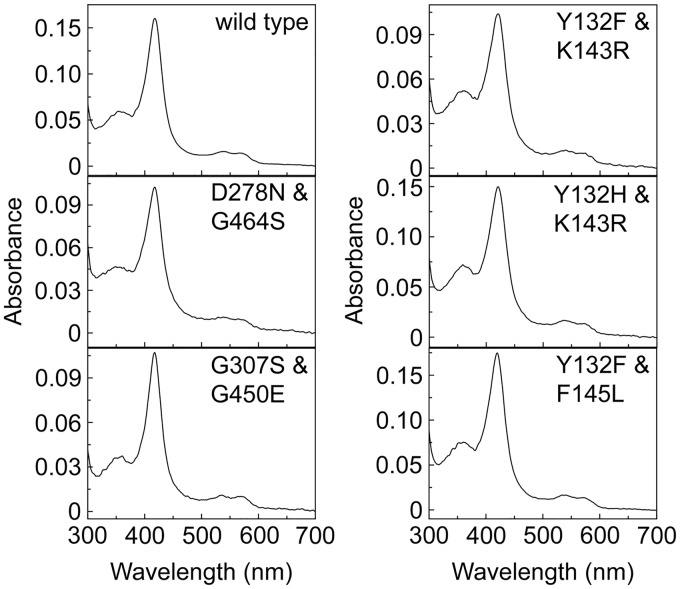
Absolute spectra of the wild-type CaCYP51 protein and CaCYP51 proteins with double amino acid substitutions. The purified CaCYP51 proteins were diluted 20-fold with 0.1 M Tris-HCl buffer (pH 8.1) and 20% glycerol, and the absolute spectra in the resting oxidized state were determined between 300 and 700 nm. The absolute spectra for the CaCYP51 proteins with single amino acid substitutions can be found in Fig. S1 in the supplemental material.

The yield of the CaCYP51 proteins after purification on Ni^2+^-nitrilotriacetic acid (NTA) agarose varied from 32 nmol per liter of culture (I471T) to 235 nmol per liter (F126V) (Table 1), indicating a 7-fold variability in expression levels in E. coli between recombinant CaCYP51 proteins. The same expression and cell breakage/solubilization conditions were used for all the CaCYP51 proteins. The CaCYP51 amino acid substitutions that gave low yields of native protein gave cell debris pellets after ultracentrifugation that were notably more red-brown in color than the pellets of the wild-type expression clone, suggesting the increased occurrence of inclusion bodies. Optimization of the expression and solubilization conditions for each CaCYP51 protein should increase native protein yields. The absolute absorbance spectra of all the CaCYP51 proteins were similar (Fig. 1; see also Fig. S1 in the supplemental material) with α, β, Soret (γ), and δ spectral bands at 566, 536, 418, and 360 nm, respectively, indicative of low-spin ferric cytochrome P450 enzymes (30, 31). Variability in the Soret peak between the CaCYP51 proteins was only ±1 nm. Dithionite-reduced carbon monoxide difference spectra for all the CaCYP51 proteins yielded a characteristic red-shifted heme Soret peak at ∼448 nm (Fig. S2), indicating that the CYP51 proteins were isolated in their native form. The main difference between the CaCYP51 proteins was the rate at which the heme Soret peak at ∼448 nm formed. There appeared to be little inactivated CaCYP51 (indicated by the appearance of a heme Soret peak at ∼420 nm for P420) present in the purified proteins. For K143R, approximately 15% of the native form of the protein was converted to the P420 inactive form after 6 min, suggesting instability in the presence of sodium dithionite.

**TABLE 1:** Yield and relative velocities of CaCYP51 proteins

CaCYP51 substitution	Yield^*a*^ (nmol liter^−1^)	Relative velocity^*b*^
Wild type	119	1.000 ± 0.047
Y118A	49	0.029 ± 0.001
F126V	235	0.298 ± 0.008
Y132F	53	0.795 ± 0.005
Y132H	28	0.135 ± 0.002
K143R	143	0.599 ± 0.010
F145L	35	0.476 ± 0.021
P230L	71	0.250 ± 0.003
Y257H	93	0.554 ± 0.029
D278N	177	0.645 ± 0.028
S279F	152	0.289 ± 0.017
G307S	193	0.031 ± 0.001
S405F	66	0.758 ± 0.011
V437I	37	0.796 ± 0.013
Y447H	36	0.965 ± 0.019
G448E	35	0.407 ± 0.007
F449Y	44	0.548 ± 0.088
G450E	163	0.517 ± 0.019
V456I	157	1.560 ± 0.018
G464S	29	0.609 ± 0.023
R467K	38	0.471 ± 0.023
I471T	32	0.634 ± 0.015
Q474K	35	1.601 ± 0.035
V488I	152	1.167 ± 0.032
Y132F+K143R	67	0.208 ± 0.014
Y132H+K143R	79	0.068 ± 0.001
Y132F+F145L	93	0.405 ± 0.012
D278N+G464S	48	0.327 ± 0.002
G307S+G450E	136	0.077 ± 0.008

a The yield of purified CaCYP51 protein isolated from 1 liter of E. coli expression culture.

b A relative velocity of 1.000 relates to a catalytic turnover number of 0.574 min^−1^ obtained with the wild-type CaCYP51 protein in the CYP51 reconstitution assay.

Catalytic turnover assayed by using lanosterol as the substrate and in the presence of a cognate human cytochrome P450 reductase (HsCPR) varied between purified CaCYP51 proteins (Table 1), with the highest turnover numbers of 0.896 and 0.919 min^−1^ being observed with the V456I and Q474K proteins, respectively. In contrast, the G307S+G450E, Y132H+K143R, G307S, and Y118A proteins had the lowest catalytic turnover numbers of 0.044, 0.039, 0.018, and 0.016 min^−1^, respectively, whereas the value was 0.574 min^−1^ for wild-type CaCYP51, using HsCPR as the redox partner. Most (20/23) enzymes with CaCYP51 single amino acid substitutions and all enzymes with double substitutions were catalytically less efficient than the wild-type enzyme (Table 1).

### Azole IC_50_ studies.

**FIG 2:**
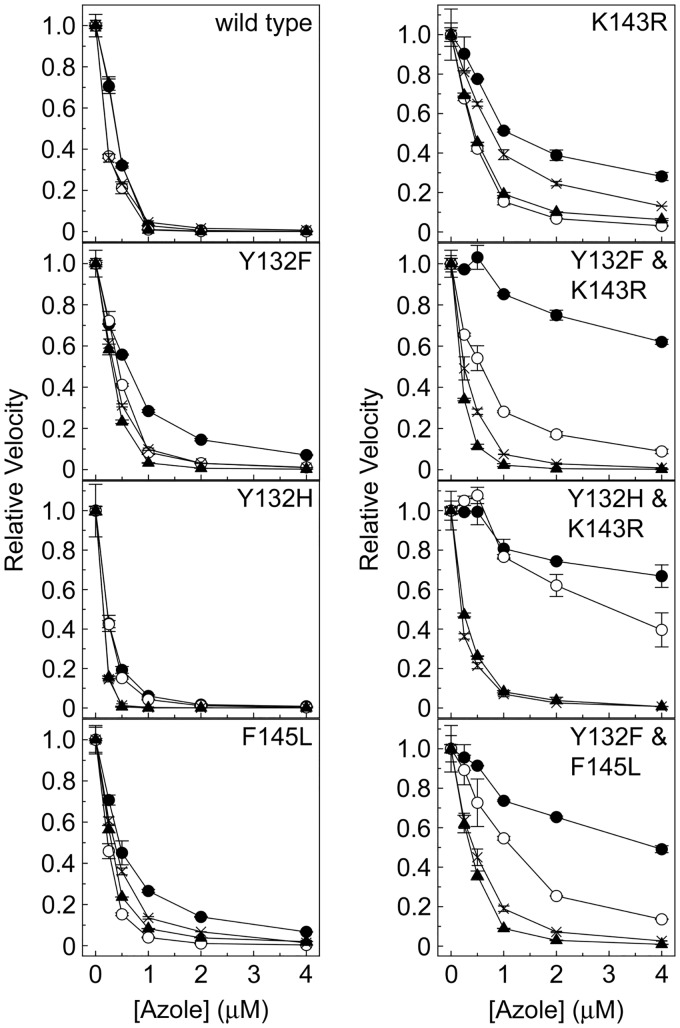
Triazole IC_50_ determinations for wild-type CaCYP51 and CaCYP51 proteins containing Y132F, Y132H, K143R, and F145L amino acid substitutions. IC_50_ values were determined for fluconazole (filled circles), voriconazole (hollow circles), itraconazole (filled triangles), and posaconazole (crosses). IC_50_ assays were performed in duplicate. Mean data points together with standard deviations (as error bars) are shown.

**FIG 3:**
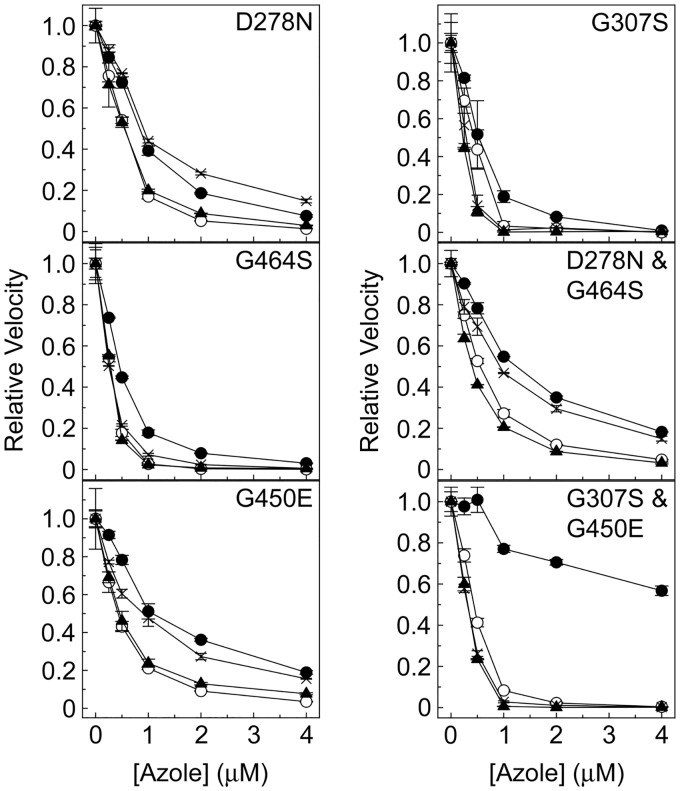
Triazole IC_50_ determinations for CaCYP51 proteins containing D278N, G307S, G450E, and G464S amino acid substitutions. IC_50_ values were determined for fluconazole (filled circles), voriconazole (hollow circles), itraconazole (filled triangles), and posaconazole (crosses). IC_50_ assays were performed in duplicate. Mean data points together with standard deviations (as error bars) are shown.

**FIG 4:**
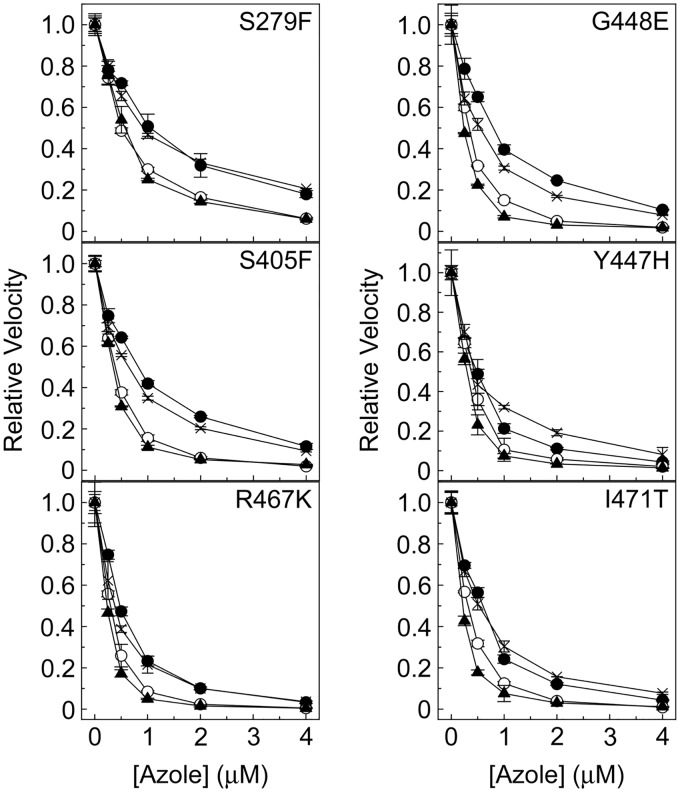
Triazole IC_50_ determinations for CaCYP51 proteins containing S279F, S405F, Y447H, G448E, R467K, and I471T amino acid substitutions. IC_50_ values were determined for fluconazole (filled circles), voriconazole (hollow circles), itraconazole (filled triangles), and posaconazole (crosses). IC_50_ assays were performed in duplicate. Mean data points together with standard deviations (as error bars) are shown.

Several CaCYP51 amino acid substitutions conferred tolerance to inhibition of *in vitro* CYP51 activity by fluconazole (Table 2). Among the single substitutions, K143R, S279F, and G450E appeared to confer the greatest catalytic tolerance to inhibition by fluconazole (Fig. 2, 3, and 4), yielding values of the concentration causing 50% enzyme inhibition (IC_50_) 3-fold higher than the IC_50_ for the wild-type enzyme. The D278N, S405F, and G448E enzymes gave fluconazole IC_50_ values 2-fold higher than the IC_50_ for the wild-type enzyme. In addition, significant residual CYP51 activities (3% to 28%) were observed in the presence of 4 μM fluconazole for the enzymes with the Y132F, K143R, F145L, Y257H, D278N, S279F, S405F, Y447H, G448E, G450E, V456I, G464S, R467K, and I471T substitutions (Table 3; Fig. S4), suggesting that these substitutions confer catalytic tolerance to fluconazole. The double substitutions conferred the greatest catalytic tolerance to fluconazole *in vitro* compared to the IC_50_ for the wild-type enzyme, with 3-, 10-, 13-, 15-, and 22-fold increased IC_50_ values for fluconazole (Table 2) being observed with the D278N+G464S, Y132F+F145L, G307S+G450E, Y132F+K143R, and Y132H+K143R enzymes, respectively. This was also reflected in the high residual CYP51 activities of 18% to 75% observed with the double substitution CaCYP51 enzymes in the presence of 4 μM fluconazole (Table 3; Fig. S4). The IC_50_ experiments indicated that nine single substitutions (Y118A, F126V, Y132H, P230L, G307S, V437I, F449Y, Q474K, and V488I) appeared to confer no increase in catalytic tolerance to fluconazole. The ineffectiveness of the V437I and V488I proteins was expected, as these substitutions occur in both azole-sensitive and azole-resistant C. albicans strains (23).

**TABLE 2:** Triazole antifungal IC_50_ values obtained with CaCYP51 proteins

CaCYP51 substitution	IC_50_^*a*^ (μM)			
	Fluconazole	Voriconazole	Itraconazole	Posaconazole
Wild type	0.384 ± 0.019	0.197 ± 0.009	0.389 ± 0.013	0.195 ± 0.009
Y118A	0.194 ± 0.010	0.202 ± 0.002	0.170 ± 0.016	0.206 ± 0.004
F126V	0.278 ± 0.027	0.341 ± 0.020	0.206 ± 0.009	0.304 ± 0.070
Y132F	0.606 ± 0.066	**0.429** ± 0.012	0.309 ± 0.003	0.343 ± 0.020
Y132H	0.220 ± 0.013	0.197 ± 0.029	0.150 ± 0.028	0.195 ± 0.003
K143R	**1.100** ± 0.313	**0.424** ± 0.035	0.450 ± 0.016	**0.789** ± 0.003
F145L	0.450 ± 0.083	0.231 ± 0.031	0.300 ± 0.013	0.359 ± 0.018
P230L	0.334 ± 0.086	0.241 ± 0.042	0.265 ± 0.038	0.238 ± 0.002
Y257H	0.512 ± 0.047	0.350 ± 0.039	0.336 ± 0.014	**0.611** ± 0.017
D278N	**0.838** ± 0.027	**0.555** ± 0.004	0.545 ± 0.044	**0.908** ± 0.020
S279F	**1.046** ± 0.325	**0.487** ± 0.002	0.569 ± 0.121	**0.912** ± 0.016
G307S	0.526 ± 0.211	**0.439** ± 0.018	0.225 ± 0.007	0.288 ± 0.042
S405F	**0.820** ± 0.042	0.362 ± 0.086	0.350 ± 0.019	**0.637** ± 0.054
V437I	0.281 ± 0.004	0.304 ± 0.002	0.293 ± 0.022	0.319 ± 0.009
Y447H	0.483 ± 0.018	0.377 ± 0.055	0.299 ± 0.039	**0.439** ± 0.135
G448E	**0.796** ± 0.087	0.339 ± 0.011	0.238 ± 0.013	**0.542** ± 0.051
F449Y	0.245 ± 0.028	0.361 ± 0.010	0.222 ± 0.011	0.366 ± 0.037
G450E	**1.078** ± 0.247	**0.427** ± 0.031	0.458 ± 0.070	**0.908** ± 0.132
V456I	0.483 ± 0.022	**0.469** ± 0.023	0.603 ± 0.002	**0.602** ± 0.017
G464S	0.455 ± 0.047	0.278 ± 0.038	0.282 ± 0.006	0.253 ± 0.024
R467K	0.476 ± 0.080	0.298 ± 0.049	0.235 ± 0.018	0.380 ± 0.011
I471T	0.600 ± 0.017	0.318 ± 0.015	0.218 ± 0.002	**0.522** ± 0.064
Q474K	0.358 ± 0.008	0.325 ± 0.002	0.246 ± 0.006	0.301 ± 0.017
V488I	0.520 ± 0.057	**0.457** ± 0.016	0.449 ± 0.016	**0.481** ± 0.052
Y132F+K143R	**5.857** ± 0.524	**0.580** ± 0.099	0.190 ± 0.002	0.246 ± 0.024
Y132H+K143R	**8.471** ± 1.271	**3.074** ± 0.042	0.237 ± 0.007	0.196 ± 0.007
Y132F+F145L	**3.889** ± 0.077	**1.158** ± 0.231	0.364 ± 0.023	**0.433** ± 0.102
D278N+G464S	**1.243** ± 0.064	**0.552** ± 0.045	0.403 ± 0.011	**0.930** ± 0.009
G307S+G450E	**4.980** ± 0.054	**0.433** ± 0.033	0.318 ± 0.017	0.312 ± 0.015

a IC_50_ determinations were performed in duplicate. Mean IC_50_ values together with standard deviations are shown. Bold values indicate IC_50_ values that are over 2-fold greater than those obtained with the wild-type protein.

**TABLE 3:** Residual CYP51 activity in the presence of 4 μM triazole antifungal

CaCYP51 substitution	Residual CYP51 activity (%) ± SD^*a*^			
	Fluconazole	Voriconazole	Itraconazole	Posaconazole
Wild type	0.11 ± 0.09	0.11 ± 0.01	0.03 ± 0.02	0.66 ± 0.02
Y118A	1.51 ± <0.01	0.28 ± 0.04	1.30 ± 0.59	0.46 ± 0.35
F126V	0.09 ± 0.06	0.19 ± 0.06	0.22 ± 0.05	0.10 ± 0.01
Y132F	**7.05** ± 0.27	1.09 ± 0.05	0.19 ± 0.04	0.93 ± <0.01
Y132H	0.86 ± 0.07	0.33 ± 0.19	0.07 ± <0.01	0.18 ± 0.06
K143R	**28.14** ± 2.04	**3.12** ± 0.27	**6.27** ± 0.51	**13.09** ± 0.02
F145L	**6.71** ± 0.15	0.37 ± 0.11	1.99 ± 0.04	1.37 ± 1.21
P230L	0.05 ± 0.03	0.13 ± 0.12	0.06 ± 0.01	0.21 ± 0.05
Y257H	**3.96** ± 0.18	0.63 ± 0.27	1.37 ± 0.28	**6.42** ± 0.59
D278N	**7.59** ± 0.58	1.34 ± 0.05	**3.00** ± 0.19	**14.82** ± 0.92
S279F	**17.99** ± 1.67	**6.15** ± 0.66	**5.97** ± 0.35	**20.64** ± 0.10
G307S	0.95 ± 0.17	0.41 ± 0.04	0.53 ± 0.21	0.71 ± 0.04
S405F	**11.56** ± 1.41	1.94 ± 0.12	2.73 ± 0.13	**9.38** ± 0.53
V437I	0.22 ± 0.06	0.02 ± <0.01	0.13 ± 0.02	0.59 ± 0.20
Y447H	**4.34** ± 0.51	2.07 ± 1.04	1.45 ± 0.06	**8.19** ± 3.58
G448E	**10.46** ± 0.20	1.89 ± 0.61	1.75 ± 0.26	**7.89** ± 0.29
F449Y	0.76 ± 0.08	0.56 ± 0.12	0.84 ± 0.57	**5.42** ± 0.04
G450E	**18.93** ± 0.94	**3.50** ± 0.17	**7.66** ± 0.54	**15.57** ± 0.21
V456I	**3.68** ± 0.95	0.79 ± 0.07	**3.21** ± 0.07	**6.66** ± 0.05
G464S	**3.05** ± 0.17	0.10 ± 0.01	0.54 ± 0.11	0.54 ± 0.09
R467K	**3.34** ± 0.61	0.52 ± 0.18	0.45 ± 0.17	**3.62** ± 0.09
I471T	**4.35** ± 0.33	0.92 ± 0.05	1.21 ± 0.01	**7.71** ± 0.58
Q474K	0.71 ± 0.08	0.34 ± 0.04	0.49 ± 0.04	**3.23** ± 0.18
V488I	1.06 ± 0.04	0.13 ± 0.08	0.98 ± <0.01	2.11 ± 0.14
Y132F+K143R	**62.04** ± 1.21	**8.86** ± 1.09	0.23 ± 0.13	0.86 ± 0.11
Y132H+K143R	**68.81** ± 5.68	**39.59** ± 8.63	0.70 ± 0.03	0.74 ± 0.05
Y132F+F145L	**49.10** ± 1.76	**13.58** ± 0.19	0.91 ± 0.05	2.64 ± 0.08
D278N+G464S	**18.26** ± 0.08	**4.79** ± 0.11	**3.19** ± 0.46	**14.97** ± 1.16
G307S+G450E	**56.75** ± 2.30	0.41 ± 0.10	0.20 ± 0.04	0.26 ± 0.11

a Residual activity is expressed as a percentage of the CYP51 activity observed in the absence of triazole antifungals. Each assay was performed in duplicate. These data are also represented graphically in Fig. S4 in the supplemental material. Residual CYP51 activities greater than 3% are indicated in bold.

Based on the IC_50_ values (Table 2), catalytic tolerance to voriconazole was less pronounced than that to fluconazole among the CaCYP51 proteins, with only 2- to 3-fold increases in IC_50_s being observed with the Y132F, K143R, D278N, S279F, G307S, G450E, V456I, Y132F+K143R, D278N+G464S, and G307S+G450E enzymes. The greatest tolerance to voriconazole was observed with the Y132F+F145L and Y132H+K143R enzymes, which gave IC_50_ values 6- and 16-fold higher than the IC_50_ observed for the wild-type CaCYP51 enzyme. In addition, residual CYP51 activities of over 3% were observed with the K143R, S279F, G450E, Y132F+K143R, Y132H+K143R, Y132F+F145L, and D278N+G464S enzymes in the presence of 4 μM voriconazole (Table 3; Fig. S4), relative to 0.11% residual activity for the wild-type enzyme.

The IC_50_ values obtained for itraconazole with the CaCYP51 proteins with amino acid substitutions ranged from 0.15 and 0.60 μM (Table 2), whereas the IC_50_ for the wild-type enzyme was 0.39 μM. However, residual CYP51 activities of 3% to 8% in the presence of 4 μM itraconazole (Table 3; Fig. S4) indicate limited catalytic tolerance to itraconazole due to the K143R, D278N, S279F, G450E, V456I, and D278N+G464S substitutions.

The IC_50_ values obtained for posaconazole varied between 0.2 μM (for the wild-type enzyme) and 0.93 μM (for the D278N+G464S enzyme) (Table 2), suggesting poor catalytic tolerance to posaconazole among the CaCYP51 proteins. Residual CYP51 activities of 3% to 21% in the presence of 4 μM posaconazole (Table 3; Fig. S4) confirmed the limited catalytic tolerance to posaconazole for the enzymes with the K143R, Y257H, D278N, S279F, S405F, Y447H, G448E, F449Y, G450E, V456I, R467K, I471T, Q474K, and D278N+G464S substitutions. P230L, previously predicted to confer itraconazole and posaconazole resistance (32, 33), did not confer catalytic tolerance to either itraconazole or posaconazole using *in vitro* CaCYP51 reconstitution assays.

### Azole ligand binding.

**FIG 5:**
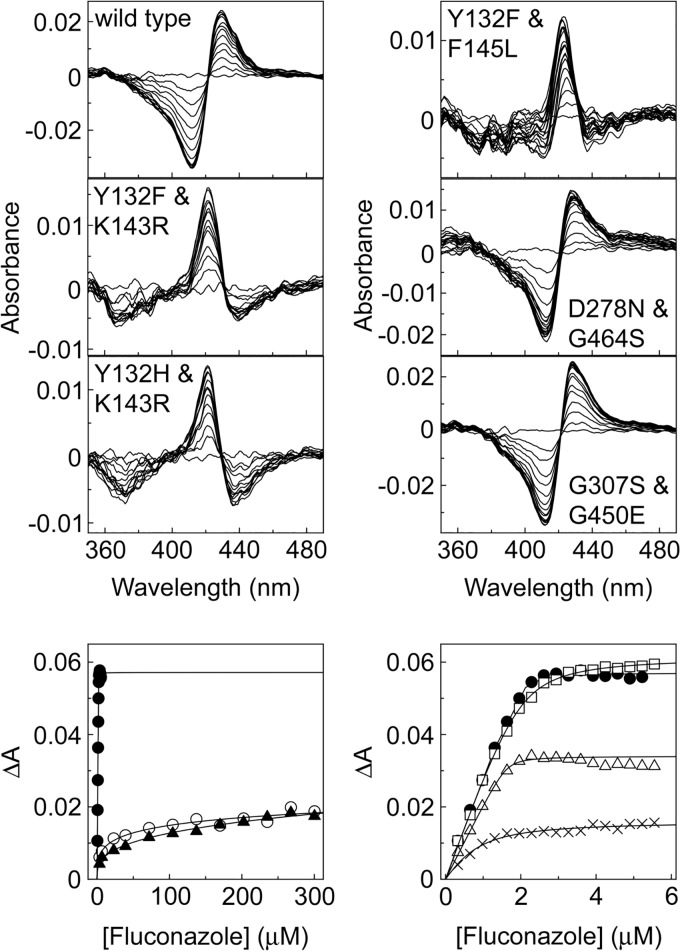
Fluconazole binding properties of CaCYP51 proteins. Type II difference spectra for 4 μM wild-type CaCYP51 and the five CaCYP51 proteins with double substitutions are shown along with the ligand binding saturation curves for the wild-type protein (filled circles) and proteins with the Y132F+K143R (hollow circles), Y132H+K143R (filled triangles), Y132F+F145L (crosses), D278N+G464S (hollow triangles), and G307S+G450E (hollow squares) substitutions. Ligand binding determinations were performed in triplicate, although only one replicate is shown. Ligand saturation curves were fitted using the modified Morrison equation (54).

Fluconazole, voriconazole, itraconazole, and posaconazole bound to all CaCYP51 proteins, producing type II difference spectra indicative of the triazole ring N-4 nitrogen coordinating as the sixth ligand with the heme iron (34) to form the low-spin CYP51-azole complex, resulting in a red shift of the heme Soret peak. The fluconazole binding difference spectra obtained with the CaCYP51 proteins with double substitutions identified a number of differences from the wild-type protein (Fig. 5). Both the D278N+G464S and G307S+G450E proteins gave type II difference spectra that were similar to the spectrum of the wild-type CaCYP51 protein with peaks at 428 to 430 nm and troughs at 412 nm. However, both the Y132F+K143R and Y132H+K143R proteins gave less intense difference spectra (change in absorbance [Δ*A*]), with a blue-shifted peak at 421 nm and a blue-shifted trough at 367 to 371 nm. Likewise, the Y132F+F145L protein gave a less intense blue-shifted spectrum, with a peak at 423 nm and a broad trough extending from 371 to 411 nm relative to the wild-type protein. These relatively weak blue-shifted type II difference spectra (Δ*A*) suggest that the Fe-N coordination between the triazole N-4 atom and the heme ferric ion has been weakened (35) because either the orientation of the triazole moiety is unfavorable or the length of the Fe-N bond has been increased. The fluconazole binding difference spectra of the enzymes with the single substitutions Y132F, Y132H, K143R, and F145L (Fig. S3) were all type II difference spectra, albeit with slightly blue-shifted peak maxima at 425 to 427 nm compared to the spectrum of the wild-type protein (429 nm). Therefore, the altered fluconazole difference spectra observed with the Y132F+K143R, Y132H+K143R, and Y132F+F145L enzymes appeared to be a cumulative effect rather than an effect attributable to one particular amino acid substitution.

Ligand binding studies with fluconazole gave dissociation constant (*K_d_*) values ranging from 7 to 90 nM for all CaCYP51 proteins with single amino acid substitutions, except for K143R (*K_d_*, 118 ± 24 [standard deviation] nM), D278N (*K_d_*, 120 ± 35 nM), G307S (*K_d_*, 133 ± 13 nM), and S279F (*K_d_*, 232 ± 36 nM). The double substitutions D278N+G464S, G307S+G450E, Y132F+F145L, Y132F+K143R, and Y132H+K143R gave *K_d_* values for fluconazole of 15 ± 9, 158 ± 38, 202 ± 57, 15,600 ± 3,900, and 18,100 ± 4,800 nM, respectively. The ligand binding studies accurately predicted the fluconazole tolerance conferred by the double substitutions Y132F+K143R and Y132H+K143R but not the catalytic tolerance to fluconazole conferred by Y132F+F145L, G307S+G450E, D278N+G464S, K143R, D278N, S279F, S405F, G448E, and G450E in the IC_50_ studies.

Preliminary ligand binding studies with voriconazole and the CaCYP51 proteins gave *K_d_* values ranging from 3 to 100 nM, with only the Y132F+K143R and Y132H+K143R enzymes having substantially higher *K_d_* values of 2,300 ± 310 and 8,000 ± 1,200 nM, respectively. Similarly, ligand binding studies for the CaCYP51 proteins with itraconazole and posaconazole gave *K_d_* values ranging from 1 to 70 nM, with the exception of those for enzymes with Y132F+K143R (*K_d_* for itraconazole, 127 ± 33 nM) and Y132H+K143R (*K_d_* for itraconazole, 387 ± 117 nM; *K_d_* for posaconazole, 162 ± 59 nM). Direct ligand binding studies with voriconazole, itraconazole, and posaconazole did not predict the observed catalytic tolerance to these three azoles conferred by several CaCYP51 amino acid substitutions in the IC_50_ experiments, as evident by the residual activities in the presence of 4 μM triazole antifungal (Table 3; Fig. S4).

### *In vitro* assessment of CaCYP51 substitutions in C. albicans.

In order to determine the impact of CYP51 amino acid substitutions on *in vitro* azole susceptibility in C. albicans, we selected several mutations representative of those that led to the greatest biochemical effect on the CYP51 enzyme. When mutant alleles were introduced into the fluconazole-susceptible parent strain SC5314, CaCYP51 amino acid substitutions displayed variable effects on the azole drug MIC (Table 4). The effects of nine single CYP51 amino acid substitutions and three double CYP51 amino acid substitutions on the fluconazole, voriconazole, itraconazole, and posaconazole MICs were tested. The G450E and K143R amino acid substitutions produced the greatest relative increase in the MIC of fluconazole, with each producing a 16-fold increase in the MIC compared to that for the reference strain, SC5314, which lacks any substitution in CYP51. The strains containing the CYP51 single amino acid substitutions Y132F, G448E, and G464S also demonstrated resistance to fluconazole, displaying an 8-fold increase in MIC over that for SC5314. The overall strongest effect on the fluconazole MIC occurred with the double substitutions Y132F+K143R and Y132F+F145L, each conferring a 32-fold increase in the fluconazole MIC. Against voriconazole, the single substitutions Y132F, K143R, F145L, and G450E had the greatest effect on the MIC, and the strongest *in vitro* effect was observed with the Y132F+K143R and Y132F+F145L double substitutions. When tested against itraconazole, most of the strains with a CYP51 substitution showed a small increase in MIC, with the strain with the strongest single amino acid substitution, K143R, displaying an MIC (0.5 μg ml^−1^) greater than that for any of the strains with the double amino acid substitutions. In contrast to the other azoles, posaconazole appeared to be very effective against all CYP51 mutant strains, as the MICs for the reference strain and all other strains tested were identical, with the exception of the strain containing the single G448E substitution, which displayed a slight 2-fold increase in MIC over that for SC5314.

**TABLE 4:** CLSI MICs of fluconazole, voriconazole, itraconazole, and posaconazole for strains containing CYP51 amino acid substitutions

CaCYP51 substitution	MIC (μg ml^−1^)			
	Fluconazole	Voriconazole	Itraconazole	Posaconazole
None^*a*^	1	≤0.03	0.06	≤0.03
Y132F	8	0.5	0.25	≤0.03
Y132H	2	0.25	0.25	≤0.03
K143R	16	0.5	0.5	≤0.03
F145L	4	0.5	0.25	≤0.03
D278N	1	≤0.03	0.125	≤0.03
S405F	4	0.25	0.25	≤0.03
G448E	8	0.25	0.25	0.06
G450E	16	0.5	0.25	≤0.03
G464S	8	0.25	0.25	≤0.03
Y132F+K143R	32	1	0.25	≤0.03
Y132F+F145L	32	1	0.25	≤0.03
D278N+G464S	8	0.25	0.25	≤0.03

a Fluconazole-susceptible parent strain SC5314.

## DISCUSSION

Previous investigations identified all the CaCYP51 mutations studied here, with the exception of V437I and V488I, as conferring azole resistance (13, 14, 21–23). While the isolated contributions of endogenously expressed CYP51 gene mutations to azole resistance have been studied in C. albicans individually (36), here we compared the azole-CYP51 binding interactions and enzyme activity of recombinant CYP51 proteins with amino acid substitutions in conjunction with the effects of those changes on azole susceptibility in C. albicans.

Amino acid substitutions associated with an increase in azole tolerance are often at the expense of a reduction in catalytic efficiency, as has been seen previously (26, 37, 38). In this study, the catalytic rate varied 56-fold between the CYP51 proteins using lanosterol as the substrate and HsCPR as the redox partner. The lowest rate was seen with the Y118A mutant enzyme (0.0164 min^−1^), and the highest was seen with the Q474K mutant enzyme (0.919 min^−1^), whereas a rate of 0.574 min^−1^ was observed with the wild-type enzyme. Only enzymes with the V456I, Q474K, and V488I substitutions gave catalytic rates greater than the rate for the wild-type enzyme (Table 1). These higher base relative velocities of the uninhibited CYP51 variants may partly explain the elevated MICs observed previously in some clinical C. albicans isolates.

Our IC_50_ studies showed that of the 23 single amino acid substitutions tested, only 6 (K143R, D278N, S279F, S405F, G448E, G450E) caused 2-fold or greater increases in the observed IC_50_ values for fluconazole (Table 2). Similarly, with voriconazole and posaconazole, greater than 2-fold increases in the IC_50_ were observed for 7 single substitutions (Y132F, K143R, D278N, S279F, G450E, V456I, and V488I) and 11 single amino acid substitutions (K143R, Y257H, D278N, S279F, S405F, Y447H, G448E, G450E, V456I, I471T, and V488I), respectively (Table 2). This was in contrast to the findings for itraconazole, where none of the substituted CaCYP51 proteins gave IC_50_ values greater than 1.6-fold that of the wild-type enzyme.

The double substitutions yielded larger increases in the observed IC_50_ values for both fluconazole and voriconazole. These increases ranged from 3.2-fold for D278N+G464S to 22-fold for Y132H+K143R with fluconazole and from 2-fold for G307S+G450E to 15.6-fold for Y132H+K143R with voriconazole, indicating a reduced affinity for fluconazole and voriconazole. These observations are consistent with the CaCYP51 structure (39), in which residues Y132, K143, G307, and S405 are exposed in the active-site cavity and substitution at these residue positions is likely to cause altered fluconazole and voriconazole binding. An increase in the IC_50_ of posaconazole was also observed with two double substitutions, Y132F+F145L (2.2-fold) and D278N+G464S (4.8-fold), in contrast to the findings for itraconazole, where no increases in IC_50_ were observed for the double substitution proteins.

The IC_50_ studies also revealed that some amino acid substitutions resulted in residual sterol demethylase activity (>3%) at high concentration of an azole (4 μM), which may result in azole tolerance. This residual activity was observed for all of the enzymes with double substitutions with fluconazole and several other mutation/azole combinations, which also exhibited greater than 2-fold changes in the IC_50_. Importantly, residual activity was also observed for 8 single substitutions (Y132F, F145L, Y257H, Y447H, V456I, G464S, R467K, and I471T) with fluconazole and 3 single substitutions (F449Y, R467K, and Q474K) with posaconazole, which did not have greater than 2-fold changes in the IC_50_. In these cases, although the IC_50_ value did not significantly increase, the residual CYP51 activity might favor the survival of the C. albicans strain with a substitution during a period of azole stress over the survival of the wild-type strain. In addition, the high level of residual activity in the double mutants, particularly with fluconazole (Table 3), further highlights the impact of a double mutation on azole resistance.

Direct azole ligand binding studies using pure recombinant CaCYP51 proteins did not always reflect the observed azole tolerance conferred by an amino acid substitution on CYP51 enzyme activity. The *K_d_* values for fluconazole for the Y132F+K143R and Y132H+K143R enzymes were 312- and 362-fold larger than the value for the wild-type enzyme, reflecting the large increases in observed IC_50_ values. However, the apparent *K_d_* values for fluconazole for the D278N+G464S enzyme was 3-fold lower than that for the wild-type enzyme, and the *K_d_* values obtained with the G307S+G450E and Y132F+F145L enzymes were only 3- and 4-fold higher than the value for the wild-type enzyme. Similarly, only the Y132F+K143R and Y132H+K143R enzymes had substantially larger *K_d_* values (2,300 and 8,000 nM, respectively) for voriconazole than the other CaCYP51 proteins (3 to 100 nM), while ligand binding experiments with itraconazole and posaconazole failed to identify any large changes in apparent *K_d_* values among the CaCYP51 proteins in this study.

Therefore, azole ligand binding studies in the absence of a substrate and a redox partner do not always identify the subtle differences between the mutant CaCYP51 proteins. While this may in part be due to the purified CaCYP51 protein adopting a conformation in free solution slightly different from that when it is partitioned into a lipid bilayer in combination with a substrate and a redox partner, it is also consistent with reports suggesting that substitutions, like G450E, that lie outside of the azole contact areas of the CYP51 molecule may contribute to azole resistance by other means, such as influencing an interaction with cytochrome P450 reductase (CPR) (39). Azole ligand binding studies are a useful tool to quickly screen new azole compounds for tight binding against a given CYP51 enzyme; however, effectiveness as a CYP51 inhibitor needs to be confirmed by IC_50_ studies.

Studies in C. albicans showed that those CYP51 mutants tested had the greatest effect on resistance to fluconazole (Table 4). This was expected, as most of the mutations are likely to have arisen in the clinic as a result of prolonged exposure to fluconazole. The high azole resistance observed with some substitutions correlated well with large changes in the IC_50_ and residual activity. All substitutions except D278N resulted in an increase in the MIC of fluconazole when tested in C. albicans, and all substitutions except Y132H and D278N resulted in the strain becoming either susceptible-dose dependent (MIC, 4 μg ml^−1^) or resistant (MIC, ≥8 μg ml^−1^) to fluconazole, according to CLSI clinical breakpoints, which are used in predicting the patient response to drug treatment (40). In particular, both the strain with the enzyme with the double substitution Y132F+K143R and the strain with the enzyme with the double substitution Y132F+F145L showed fluconazole MIC changes of 32-fold, in accordance with their high IC_50_s and residual activities. Strains with enzymes with D278N+G464S, Y132F+K143R, and Y132F+F145L showed voriconazole MIC changes of 8-, 32-, and 32-fold, respectively, compared with that for the susceptible SC5314 isolate, which correlates with their high residual CYP51 activities.

Posaconazole was an effective inhibitor of all the C. albicans strains tested, despite the fact that most of the CaCYP51 amino acid substitutions gave increased IC_50_s and relatively high residual demethylation activity. This is in agreement with the findings of previous studies where multiple CaCYP51 amino acid substitutions tested in Saccharomyces cerevisiae (including S405F, Y132H, and G464S) did not affect posaconazole MICs (41). It was also shown that in C. albicans clinical matched isolates, CYP51 substitutions also had a minimal impact on posaconazole susceptibility, except for the strain with the combination of Y132H and G464S mutations (41). Importantly, this means that posaconazole remains an effective agent against the fluconazole-resistant CaCYP51 strains tested here.

None of the mutant CaCYP51 proteins yielded an IC_50_ value for itraconazole that was greater than 1.6-fold that for the wild-type CaCYP51 enzyme, although the itraconazole MIC for the C. albicans mutant strain with the K143R CYP51 substitution was increased above the susceptible dose-dependent CLSI breakpoint of 0.25 μg ml^−1^, which correlates with the 6.3% residual CYP51 activity observed with the recombinant CaCYP51 protein with K143R at 4 μM itraconazole. All other tested C. albicans strains (those with the Y132F, Y132H, F145L, D278N, S405F, G448E, G450E, G464S, Y132F+K143R, Y132F+F145L, and D278N+G464S substitutions) caused small, albeit consistent, increases in the itraconazole MIC over that for strain SC5314. However, these results do not support the previous observations that some amino acid substitutions, including P230L, Y447H, and G450E, conferred itraconazole resistance in clinical C. albicans strains (23). In these cases, there may have been other resistance mechanisms involved.

In some cases, there was little correlation between the IC_50_ and the MIC observed. The individual Y132H, F145L, and G464S substitutions conferred approximately 2-, 4-, and 5-fold increases in the MIC of fluconazole, respectively, for the mutant strains over that for SC5314, despite the IC_50_ for the Y132H protein being less than that for the wild-type CYP51 and the F145L and G464S substitutions conferring only slightly increased catalytic tolerance to fluconazole. The apparent ineffectiveness of several point mutations that have previously been associated with fluconazole resistance (especially Y132H, which was one of the first substitutions identified in resistant strains; F145L; and G464S) was surprising. However, Kudo et al. (26) and Bellamine et al. (27) established, using *in vitro* IC_50_ studies, that F145L contributed the majority of the increase in azole tolerance observed in the mutant with the double substitution Y132H+F145L.

Similarly, the Y132F, K143R, F145L, and G450E CYP51 mutants showed a 16-fold increase in voriconazole MIC over that for susceptible isolate SC5314, and the Y132H, S405F, G448E, and G464S CYP51 mutants also showed 8-fold increases in the MIC for voriconazole, despite less than 3-fold increases in the IC_50_ for voriconazole being observed with the CaCYP51 enzymes with single substitutions.

These findings for the C. albicans strains suggest that these mutations may reduce susceptibility by means not detected in our CYP51 reconstitution assays. Therefore, it can be difficult to demonstrate a direct correlation between the observed phenotype (azole resistance) of a C. albicans strain and a direct causal effect of a single amino acid substitution in the CaCYP51 enzyme using the biochemical tools used in the present study. It is plausible that single substitutions which do not confer remarkably high residual activities, IC_50_ values, or relative velocities (such as Y132H, P230L, G307S, and F449Y) may affect cell tolerance to azoles in ways not measured here.

In summary, the greatest variation in azole susceptibility among the proteins with CaCYP51 amino acid substitutions was observed for fluconazole, with the mutants with double substitutions being particularly resistant to inhibition by fluconazole. This confirms the finding that the cumulative inhibitory effect of additional substitutions in CaCYP51 is greater than the effect of individual mutations in isolation and accounts for the common occurrence of multiple amino acid substitutions in the CYP51 genes isolated from azole-resistant C. albicans strains. The lesser effects against itraconazole and posaconazole observed support the suggestion that these agents are plausible alternative therapies against CYP51-mediated fluconazole-resistant C. albicans. This observation is consistent with both the C. albicans and Saccharomyces cerevisiae CYP51 structures (39, 42), which predict that longer drug molecules generally correlate with a greater potency for inhibition of fungal CYP51s. These crystal models show the longer posaconazole and itraconazole molecules forming additional contacts with the amino acids that line the substrate access channel, so stabilizing the closed (drug-bound) conformation of the protein, whereas the smaller fluconazole and voriconazole molecules interact only with the exposed amino acids in the active-site pocket and the heme. Even so, the possibility that prolonged exposure to other azole agents might select for additional azole-specific CYP51 gene mutations in C. albicans must be considered. In agriculture, complex genotypes with multiple substitutions have been selected during the changing regimes of azole fungicides deployed over recent decades, and in some countries, wild-type alleles of CYP51 are not seen (43). Nevertheless, fungal phytopathogens have been managed, and we can hope for similar responses to the changing genotypes of CYP51 in Candida albicans.

## MATERIALS AND METHODS

### Construction of C. albicans CYP51 mutant strains.

The strains used in this study are shown in Table 5. Strains expressing seven single mutations (Y132F, K143R, F145L, S405F, G448E, G450E, G464S) and three double mutations (Y132F+K143R, Y132F+F145L, D278N+G464S) were selected from a previous study (36). Two additional strains expressing the Y132H and D278N CYP51 gene mutations were created utilizing the *SAT1*/FLP cassette described previously (44). Briefly, C. albicans isolate SC5314 was transformed via electroporation with inserts of a pSFS2-derived plasmid possessing the *SAT1* nourseothricin resistance marker, FLP recombinase, and mutant CaCYP51 genes of interest. The 5′ end of the DNA fragment contained either the Y132H or D278N CYP51 open reading frame (ORF), and the 3′ end possessed homology downstream of the CaCYP51 stop codon to ensure homologous recombination at the CYP51 gene locus. Successful transformants were screened on yeast-peptone-dextrose agar plates containing nourseothricin and confirmed via Southern hybridization. Genomic DNA from nourseothricin-screened transformants was digested with the HindIII restriction enzyme, and the resulting DNA digests were loaded on 1% agarose gels containing ethidium bromide. Digested samples were visualized briefly under UV light after approximately 2 h of electrophoresis (120 V) to confirm appropriate band separation. Lanes containing digested DNA in the gel were transferred onto a nitrocellulose membrane via vacuum suction, and DNA was cross-linked to the membrane through UV exposure. PCR amplification of the genomic DNA from parent isolate SC5314 using primers ERG11_Probe_F (5′-AGTTCAATGGTGGTTTTACCTACT-3′) and ERG11_Probe_R (5′-ATTTCTGATTGAGTCATCCTAACA-3′) generated a 254-bp product, used as a probe for the *ERG11* gene locus. The probe was labeled and prepared using an Amersham AlkPhos direct labeling and detection system (GE Life Sciences) and hybridized to the membrane through overnight incubation at 55°C. Bands containing the hybridized, alkaline phosphatase-labeled probe corresponding to either wild-type *ERG11* alleles (2,678 bp) or successfully replaced alleles with the *SAT1*/FLP cassette included (4,318 bp) or recycled (3,119 bp) were visualized with the CDP-*Star* chemiluminescent detection reagent. To confirm the sequence of the replaced *ERG11* allele, a DNA fragment containing the *ERG11* ORF and approximately 435 bp upstream of the start codon and 229 bp downstream of the *ERG11* stop codon was PCR amplified using primers CaERG11_A_(ApaI) (5′-GGGCCCGGGTTATTTGAGAACAGCC-3′) and CaERG11_E_(XhoI) (5′-CTCGAGCCAGTGGACAAAAACCATCA-3′). The resulting DNA fragment was purified and Sanger sequenced on an ABI model 3130XL genetic analyzer with the following sequencing primers: CaERG11SeqA (5′-GCCACCACACCCTATGGCTATT-3′), CaERG11SeqB (5′-TATTTTCACTGCTTCAAGATCT-3′), CaERG11SeqC (5′-CCAAAAGGTCATTATGTTTTAG-3′), CaERG11SeqD (5′-CATACAAGTTTCTCTTTTTTCC-3′), CaERG11SeqE (5′-CATTTAGGTGAAAAACCTCATT-3′), and CaERG11SeqF (5′-TACTCCAGTTTTCGGTAAAGGG-3′).

**TABLE 5:** Strains used in this study

Strain	Genotype	Source or reference
SC5314	*ERG11-1/ERG11-2*	ATCC
Constructed laboratory strains^*a*^		
20E1II1G1	*ERG11^Y132F^*::*FRT/ERG11^Y132F^*::*FRT*^*b*^	36
SCERG11R1S1C1	*ERG11^Y132H^*::*FRT/ERG11^Y132H^*::*FRT*	This study
10B1A32A	*ERG11^K143R^*::*FRT/ERG11^K143R^*::*FRT*	36
2A1A18A	*ERG11^F145L^*::*FRT/ERG11^F145L^*::*FRT*	36
SCERG11R3S3C1	*ERG11^D278N^*::*FRT/ERG11^D278N^*::*FRT*	This study
21C1M1A1	*ERG11^S405F^*::*FRT/ERG11^S405F^*::*FRT*	36
20NA11A57A	*ERG11^G448E^*::*FRT/ERG11^G448E^*::*FRT*	36
15A3A108A	*ERG11^G450E^*::*FRT/ERG11^G450E^*::*FRT*	36
19A1A1C1	*ERG11^G464S^*::*FRT/ERG11^G464S^*::*FRT*	36
9A14A21	*ERG11^Y132F,K143R^*::*FRT/ERG11^Y132F,K143R^*::*FRT*	36
27A5A33A	*ERG11^Y132F,F145L^*::*FRT/ERG11^Y132F,F145L^*::*FRT*	36
13A1A57A	*ERG11^D278N,G464S^*::*FRT/ERG11^D278N,G464S^*::*FRT*	36

a All laboratory strains have SC5314 as the background.

b FRT, FLP recombination target.

### Susceptibility testing of C. albicans CYP51 mutant strains.

Azole MICs were determined using the broth microdilution method described by the Clinical Laboratory and Standards Institute (45, 46). Cells were incubated in 96-well microtiter plates containing RPMI 1640 medium and serially diluted concentrations of either fluconazole, itraconazole, voriconazole, or posaconazole. The concentrations for MIC determinations ranged from 0.125 to 64 μg ml^−1^ for fluconazole and 0.03 to 16 μg ml^−1^ for itraconazole, voriconazole, and posaconazole. Measurements were read visually at 24 h after incubation at 35°C for a 50% reduction in growth from that in the drug-free control wells. MIC measurements were performed in duplicate, and the MIC was reported as the higher of two values for all strains tested. In cases where the duplicate MIC values were not identical, the higher of the two MIC values is reported. Overall, the MIC values were consistent with 92% (184/200) of the MICs being within a single dilution of each other for a given strain and drug combination. Additionally, no MICs differed by more than 2 dilutions for a given strain and drug.

### Construction of *pCWori^+^*:*CaCYP51* expression vectors for expression in E. coli.

Wild-type C. albicans CYP51 (UniProtKB accession number P10613) and 28 mutant C. albicans CYP51 genes, 23 coding for single amino acid substitutions and 5 coding for double substitutions, were synthesized by Eurofins-MWG-Operon (Ebersberg, Germany) and inserted into a shuttle vector with kanamycin selection using NdeI and HindIII cloning sites flanking the start and stop codons, respectively. All the CaCYP51 genes were modified so that the first 8 amino acids encoded MALLLAVF to facilitate expression in E. coli (47), and a 12-base sequence (CATCACCATCAC) encoding a 4-histidine tag was inserted immediately in front of the stop codon to facilitate protein purification by Ni^2+^-NTA agarose affinity chromatography. All 29 genes were codon optimized for expression in E. coli.

The CaCYP51 genes were excised by digestion with NdeI and HindIII, gel purified, and then ligated into the *pCWori*^+^ expression vector that had previously been cut with NdeI and HindIII. Positive recombinants were isolated by transformation into E. coli strain DH5α and selected on LB agar plates containing 0.1 mg ml^−1^ sodium ampicillin. The sequence integrity of each *pCWori^+^*:*CaCYP51* construct was checked by DNA sequencing in both directions (Eurofins-MWG-Operon) at this stage and then again from a plasmid preparation derived from the overnight cultures used to inoculate the protein expression culture flasks.

The CaCYP51 single amino acid substitutions synthesized were Y118A, F126V, Y132F, Y132H, K143R, F145L, P230L, Y257H, D279N, S279F, G307S, S405F, V437I, Y447H, G448E, F449Y, G450E, V456I, G464S, R467K, I471T, Q474K, and V488I, and the double substitutions were Y132F+K143R, Y132H+K143R, Y132F+F145L, D278N+G464S, and G307S+G450E.

### Heterologous expression in E. coli and isolation of recombinant CaCYP51 proteins.

The *pCWori^+^*:*CaCYP51* constructs were transformed into competent E. coli DH5α cells, and transformants were selected using 0.1 mg ml^−1^ sodium ampicillin. One-liter volumes of Terrific broth (1.2% tryptone, 2.4% yeast extract, 0.4% glycerol, 0.09 M potassium phosphate, pH 7.5) containing 0.1 mg ml^−1^ sodium ampicillin in 2-liter plastic baffled flasks were inoculated with 20 ml of an overnight culture of the expression clones. The cultures were shaken at 200 rpm and 37°C for 7 h, followed by induction with 1 mM isopropyl β-d-1-thiogalactopyranoside and 1 mM the heme precursor 5-aminolevulinic acid, prior to shaking for a further 16 h at 160 rpm and 27°C. Protein isolation was done according to the method of Arase et al. (48), except that 2% sodium cholate was used in the sonication buffer. The solubilized CYP51 proteins were purified by affinity chromatography using Ni^2+^-NTA agarose as previously described (30), with the modification that 0.1% l-histidine in 0.1 M Tris-HCl (pH 8.1) and 25% glycerol were used to elute nonspecifically bound E. coli proteins after the salt washes. The CaCYP51 proteins were recovered by elution with 1% l-histidine in 0.1 M Tris-HCl (pH 8.1) and 25% glycerol, followed by dialysis against 5 liters of 25 mM Tris-HCl (pH 8.1) and 10% glycerol. The Ni^2+^-NTA agarose-purified CaCYP51 proteins were used for all subsequent spectral and IC_50_ determinations. Protein purity was assessed by SDS-polyacrylamide gel electrophoresis.

### Determination of cytochrome P450 protein concentrations.

Reduced carbon monoxide difference spectra (49) were used to determine the cytochrome P450 concentration, with carbon monoxide being passed through the cytochrome P450 solution prior to addition of sodium dithionite to the sample cuvette. An extinction coefficient of 91 mM^−1^ · cm^−1^ (50) was used to calculate the cytochrome P450 concentrations from the absorbance difference between 445 to 447 nm and 490 nm. Absolute spectra at wavelengths between 700 and 300 nm were determined using 20-fold dilutions of the purified CaCYP51 proteins in 0.1 M Tris-HCl (pH 8.1) and 20% glycerol as previously described (30). Confirmation that the isolated CaCYP51 proteins were active was demonstrated by measuring the 14α-demethylation of lanosterol using the CYP51 reconstitution assay detailed below.

### CYP51 reconstitution assay system.

IC_50_ determinations were performed using the CYP51 reconstitution assay system (final reaction volume, 500 μl) previously described (51) containing 1 μM CaCYP51, 2 μM Homo sapiens cytochrome P450 reductase (UniProtKB accession number P16435), 50 μM lanosterol, 50 μM *sn*-dilaurylphosphatidylcholine, 4% (wt/vol) 2-hydroxypropyl-β-cyclodextrin, 0.4 mg ml^−1^ isocitrate dehydrogenase, 25 mM trisodium isocitrate, 50 mM NaCl, 5 mM MgCl_2_, and 40 mM MOPS (morpholinepropanesulfonic acid; pH ∼7.2). Azole antifungal agents were added in 2.5 μl dimethyl sulfoxide, followed by 10 min of incubation at 37°C prior to assay initiation with 4 mM β-NADPH-Na_4_. The samples were then shaken for 15 min at 37°C. Sterol metabolites were recovered by extraction with ethyl acetate, followed by derivatization with 0.1 ml *N*,*O*-bis(trimethylsilyl)trifluoroacetamide (BSTFA)–trimethylchlorosilane (TMCS) (99:1) and 0.3 ml anhydrous pyridine (2 h at 80°C), prior to analysis by gas chromatography-mass spectrometry (GC-MS) (52). IC_50_ determinations were performed in duplicate.

### Azole binding studies.

Studies of azole antifungal binding to 4 μM CaCYP51 proteins were performed as previously described (53) using quartz split cuvettes with a 4.5-mm light path. Stock 1-, 0.5-, 0.2-, and 0.1-mg ml^−1^ solutions of fluconazole, itraconazole, voriconazole, and posaconazole were prepared in dimethyl sulfoxide. Azole antifungals were progressively titrated against the CaCYP51 proteins in 0.1 M Tris-HCl (pH 8.1) and 25% glycerol at 22°C, with equivalent volumes of dimethyl sulfoxide also being added to the CYP51-containing compartment of the reference cuvette. The absorbance difference spectra between 500 and 350 nm were determined after each incremental addition of azole, with binding saturation curves constructed from the change in the peak and trough absorbance (Δ*A*_peak-trough_) against the azole concentration. The dissociation constant (*K_d_*) of the enzyme-azole complex for each azole was determined by nonlinear regression (Levenberg-Marquardt algorithm) using a rearrangement of the Morrison equation for tight ligand binding (54, 55). Tight binding is normally observed when the *K_d_* for a ligand is similar to or lower than the concentration of the enzyme present (56). Where ligand binding was weak, the Michaelis-Menten equation was used to fit the data. Each binding determination was performed in triplicate. The chemical structures of the azole antifungals used in this study are shown in Fig. S5 in the supplemental material.

### Data analysis.

Curve fitting of the ligand binding data was performed using the computer program ProFit (version 6.1.12; QuantumSoft, Zurich, Switzerland). Spectral determinations were made using quartz semimicrocuvettes with a Hitachi U-3310 UV/visible spectrophotometer (San Jose, CA).

### Chemicals.

All chemicals, including all azole antifungals except voriconazole, were obtained from Sigma Chemical Company (Poole, UK). Voriconazole was supplied by Discovery Fine Chemicals (Bournemouth, UK). Growth media, sodium ampicillin, IPTG, and 5-aminolevulenic acid were obtained from Foremedium Ltd. (Hunstanton, UK). The Ni^2+^-NTA agarose affinity chromatography matrix was obtained from Qiagen (Crawley, UK).

## Supplementary Material

Supplemental file 1
