# Supplementary material for: The Evolution of Azole Resistance in Candida albicans Sterol 14α-Demethylase (CYP51) through Incremental Amino Acid Substitutions
Source: Antimicrob Agents Chemother. 2019 Apr 25;63(5):e02586-18. doi: 10.1128/AAC.02586-18 (PMC6496074; doi:10.1128/AAC.02586-18)

Figure S1 Absolute spectra of single amino acid substitution CaCYP51 proteins (panel A).

Purified CaCYP51 proteins were diluted 20-fold with 0.1 M Tris-HCl buffer (pH 8.1) and 20% glycerol. The absolute spectra in the resting oxidized state were then determined between 300 and 700 nm.

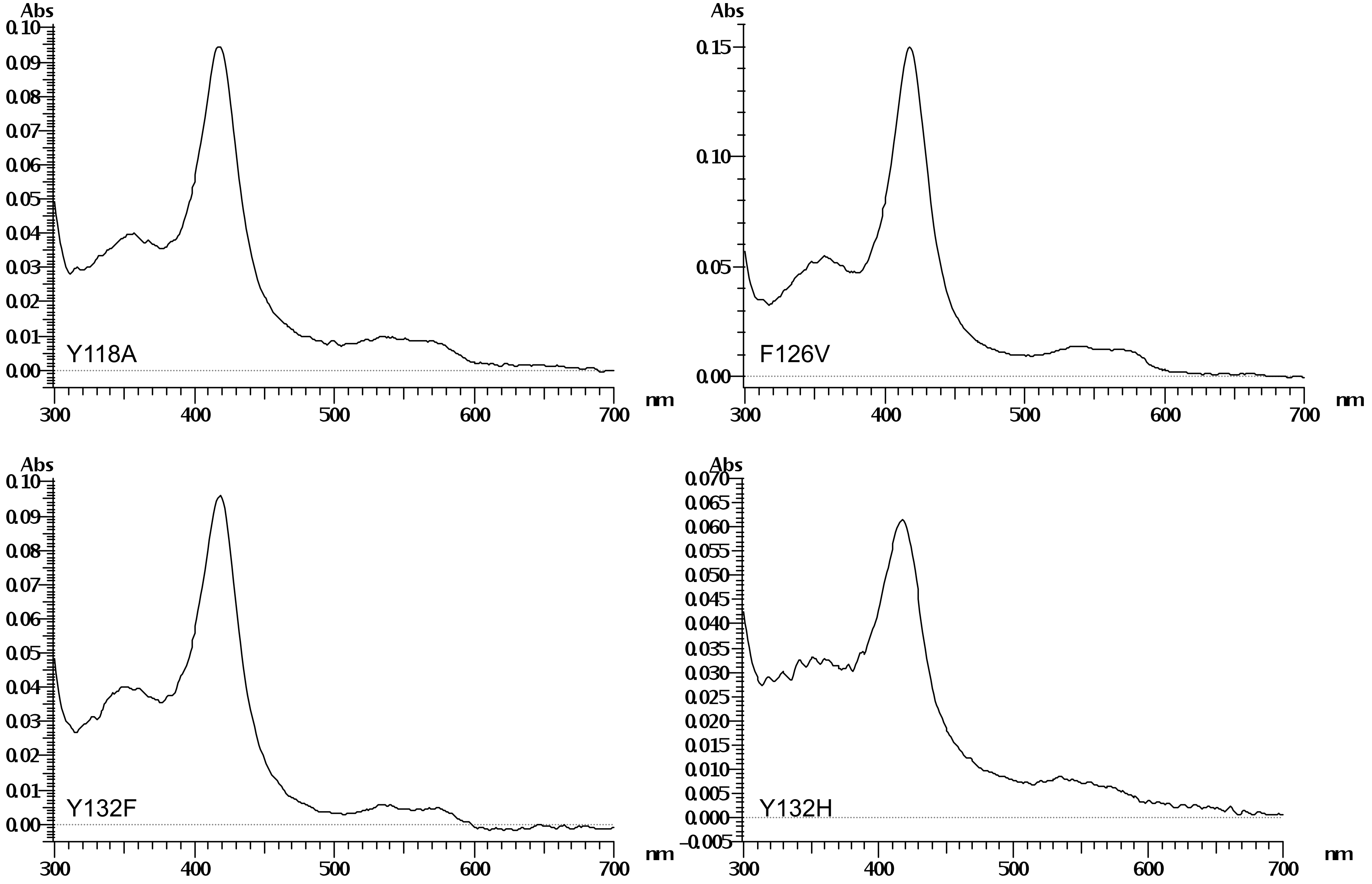

Figure S1 Absolute spectra of single amino acid substitution CaCYP51 proteins (panel B).

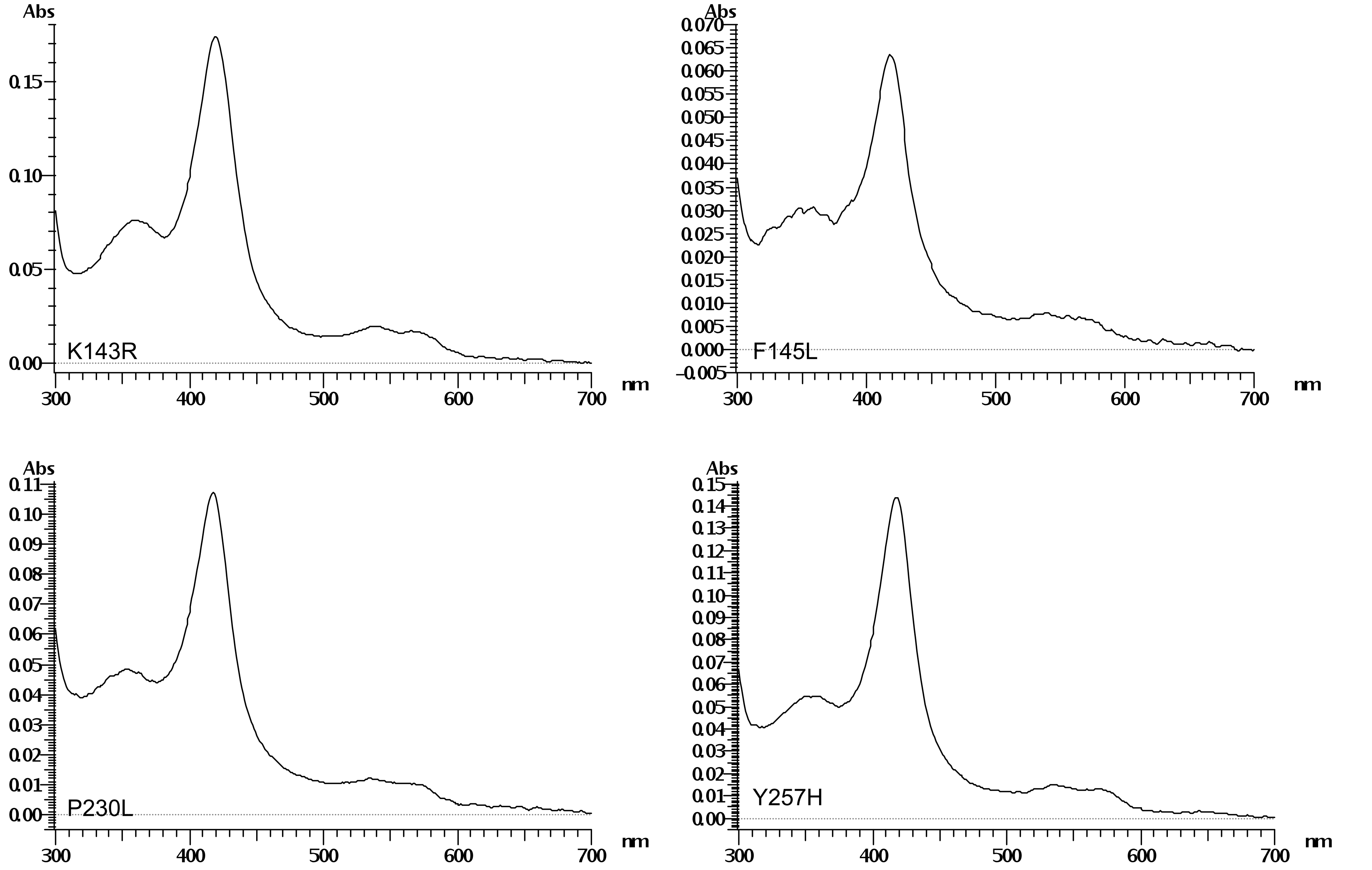

Figure S1 Absolute spectra of single amino acid substitution CaCYP51 proteins (panel C).

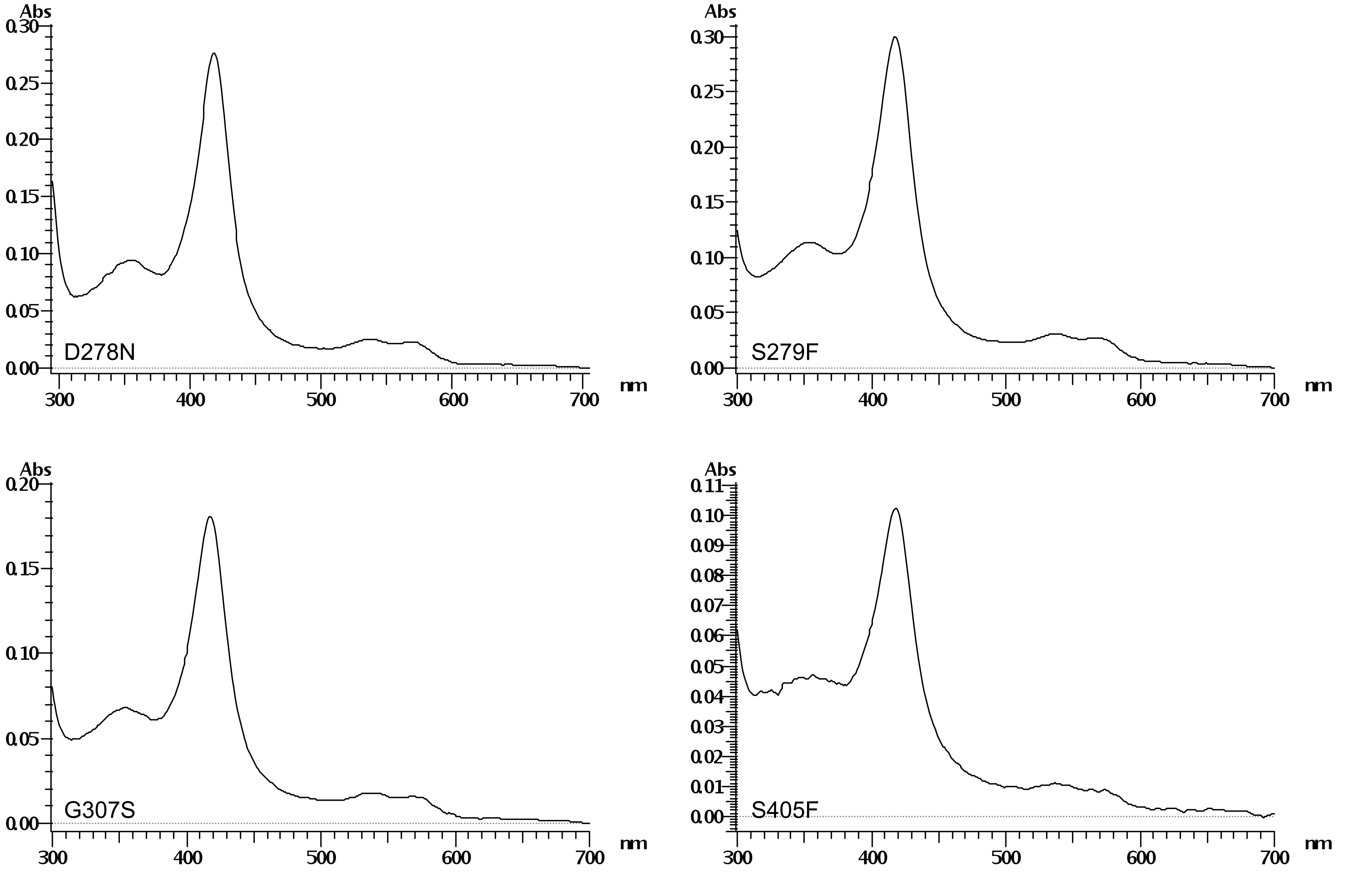

Figure S1 Absolute spectra of single amino acid substitution CaCYP51 proteins (panel D).

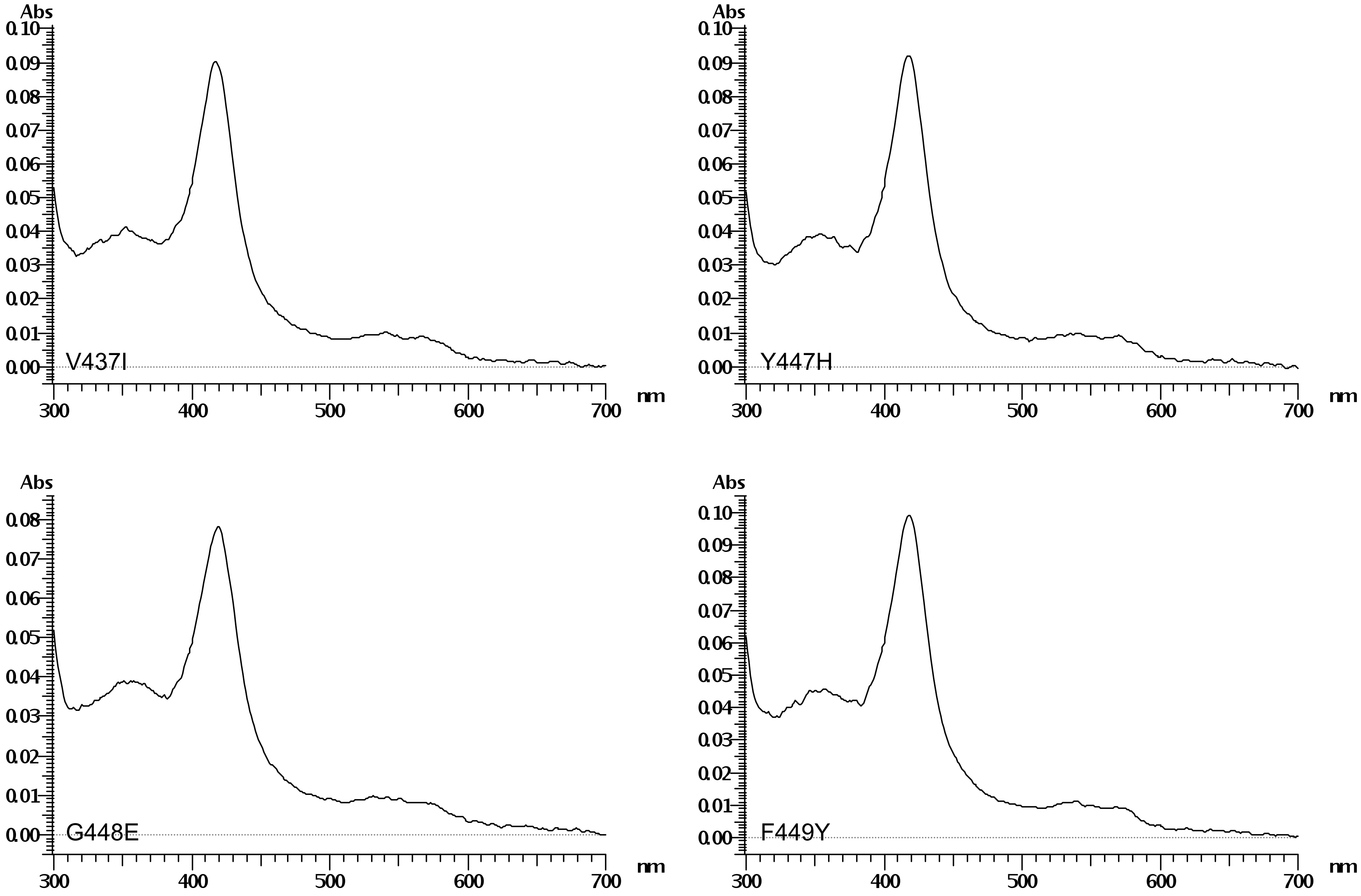

Figure S1 Absolute spectra of single amino acid substitution CaCYP51 proteins (panel E).

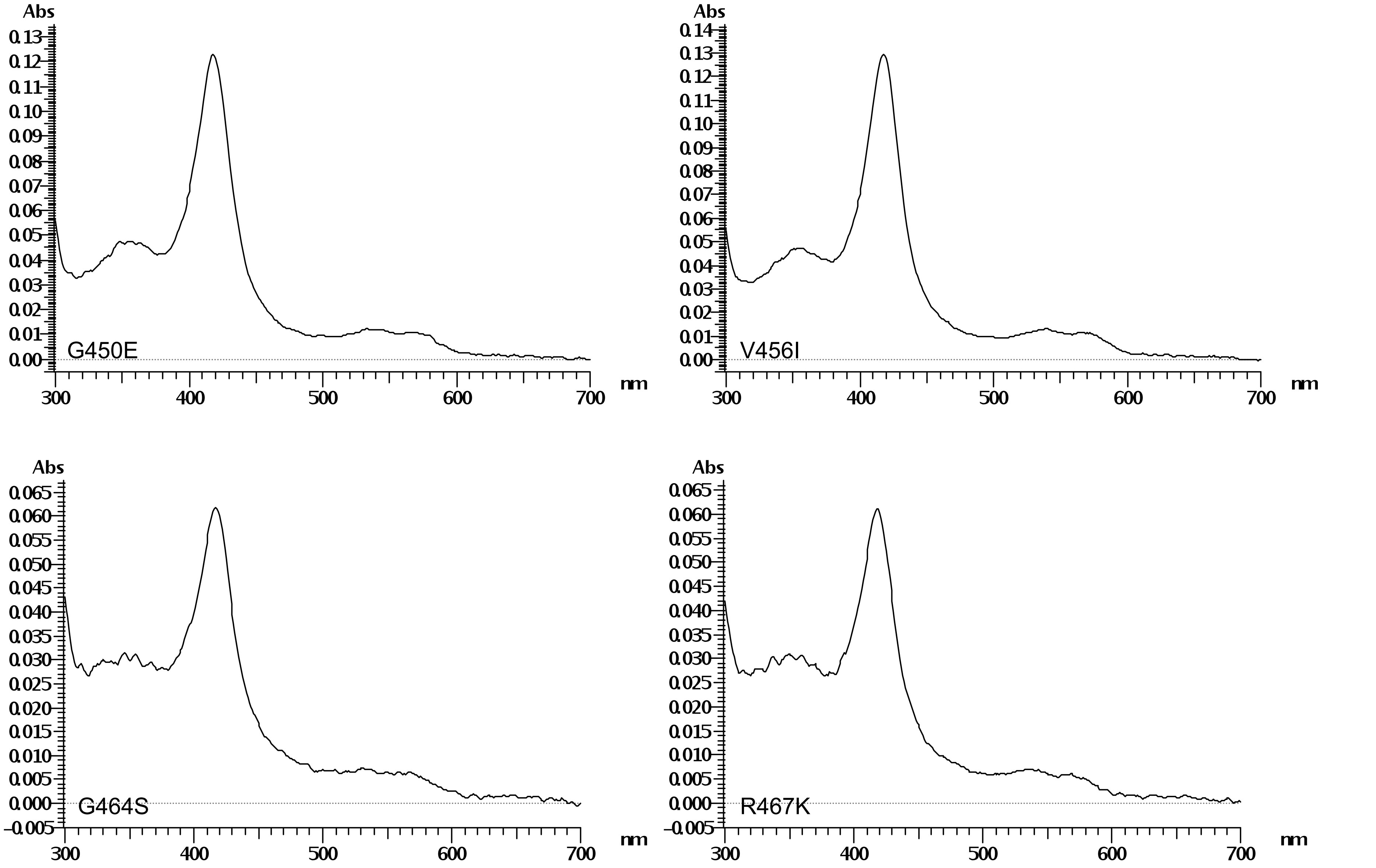

Figure S1 Absolute spectra of single amino acid substitution CaCYP51 proteins (panel F).

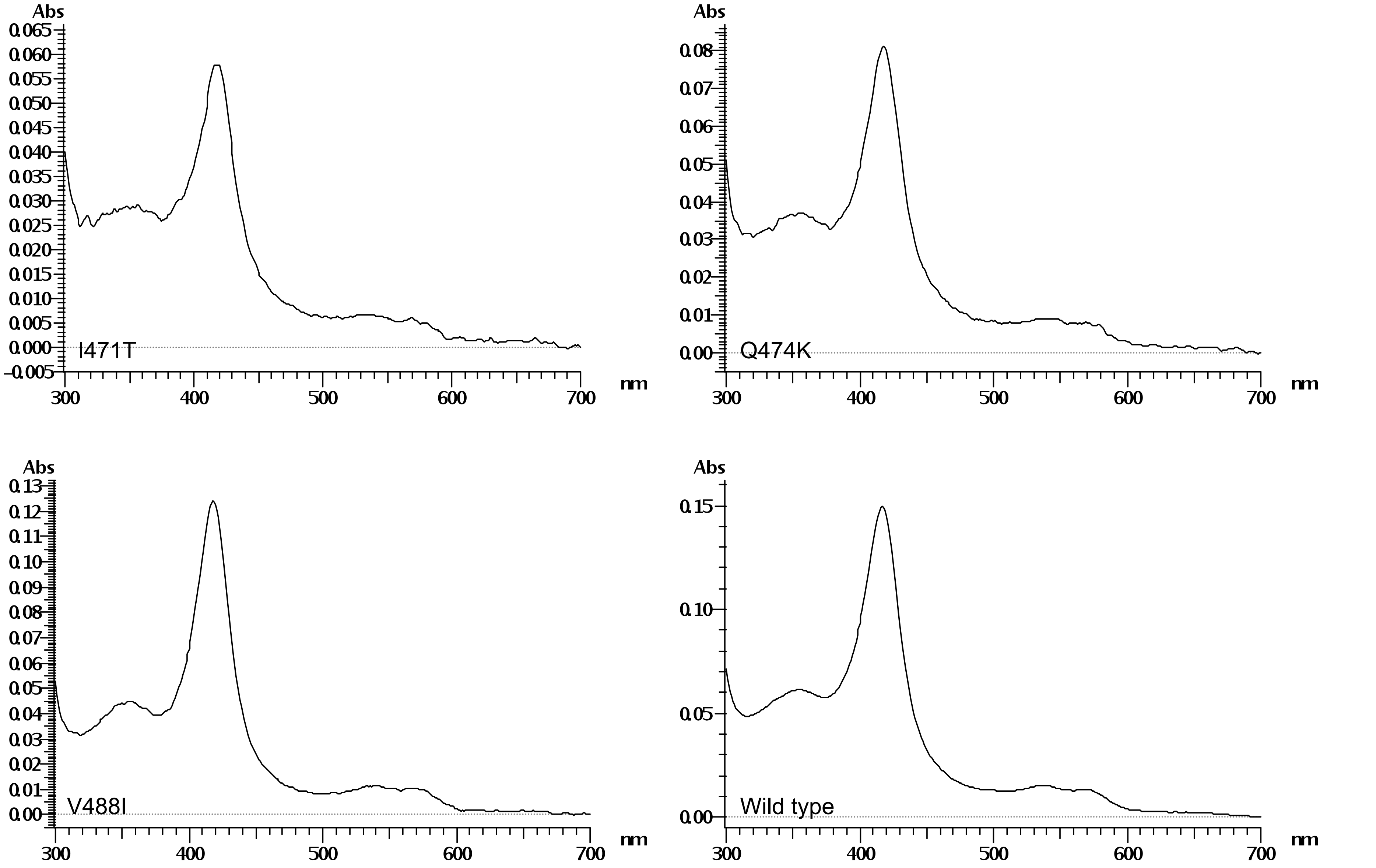

Figure S2 Dithionite-reduced carbon monoxide difference spectra of CaCYP51 proteins (panel A).

Purified CaCYP51 proteins were diluted 10-fold with 0.1 M Tris-HCl buffer (pH 8.1) and 20% glycerol. Carbon monoxide was gently passed through the CaCYP51 sample for 60 seconds prior to the sample being split between sample and reference quartz semi-micro cuvettes (10 mm light path). A background scan was then performed prior to a few grains of sodium dithionite being added to the sample cuvette and the contents mixed with a glass Pasteur pipette. Sequential wavelength scans between 500 and 400 nm were then performed at 45 second intervals until the emergent red-shifted CO-P450 heme Soret peak at ~448 nm reached a maximum.

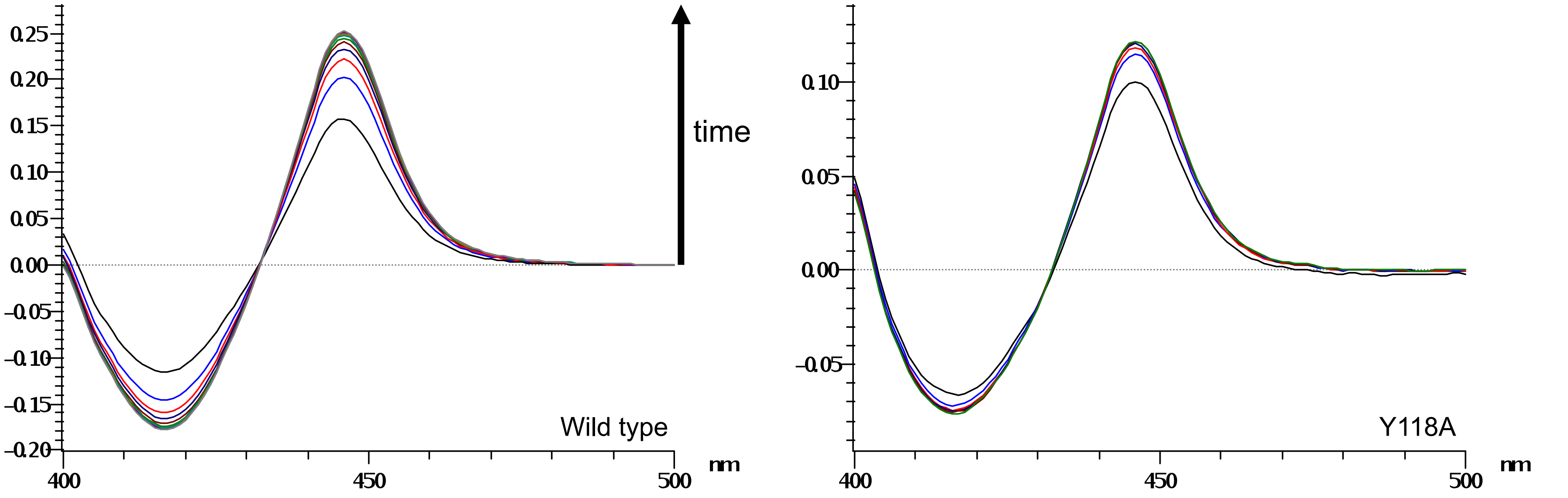

Figure S2 Dithionite-reduced carbon monoxide difference spectra of CaCYP51 proteins (panel B).

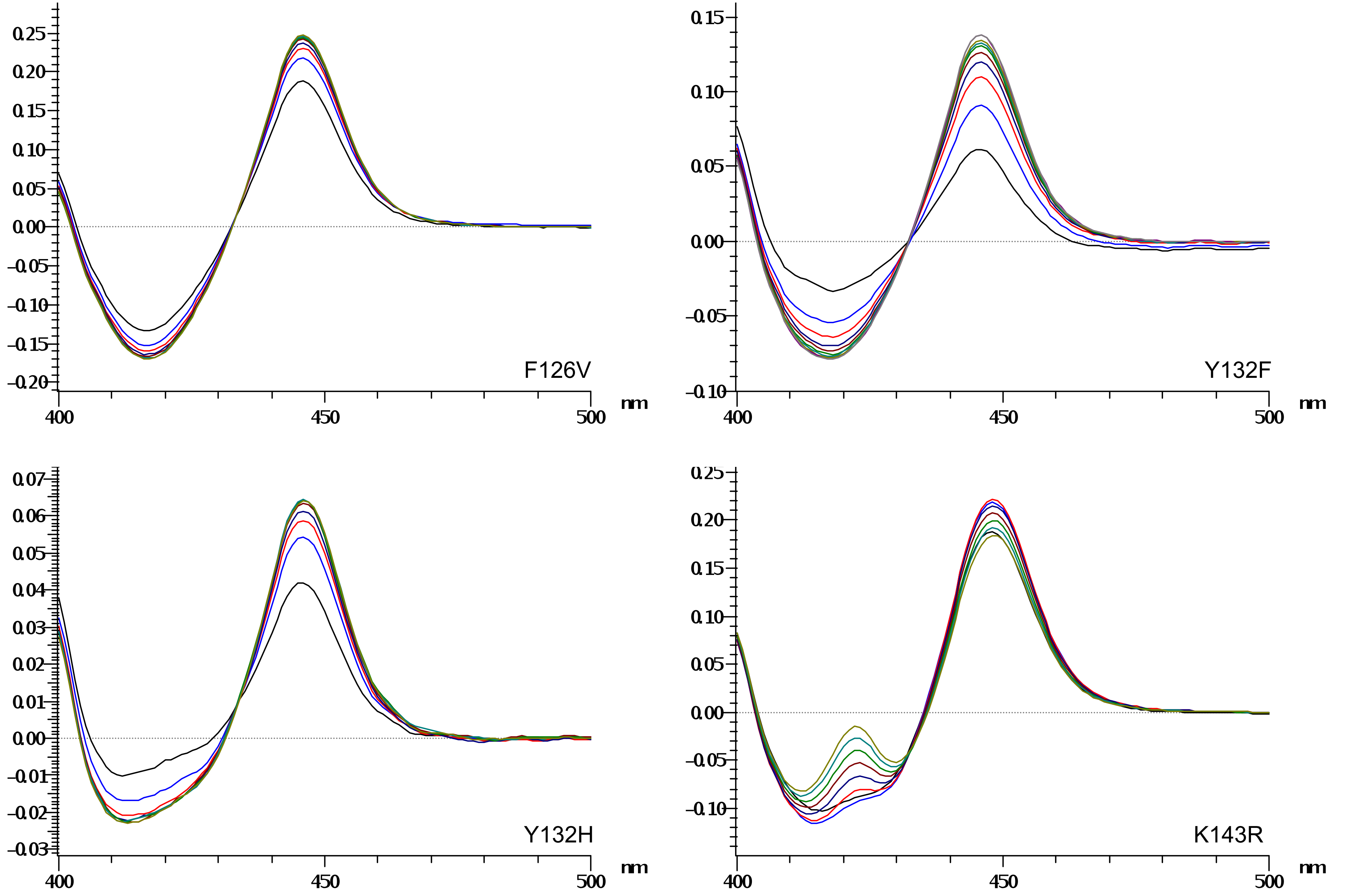

Figure S2 Dithionite-reduced carbon monoxide difference spectra of CaCYP51 proteins (panel C).

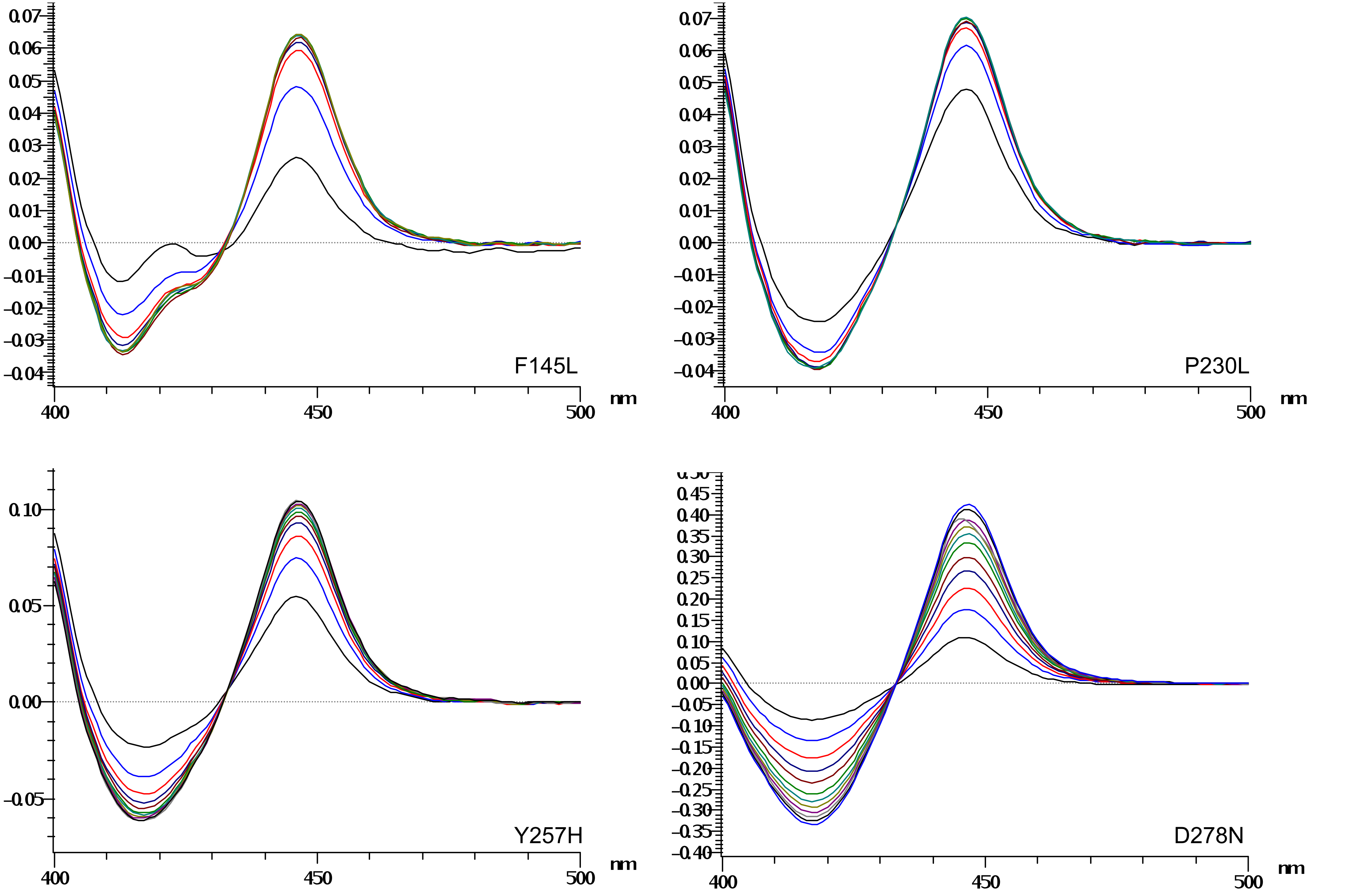

Figure S2 Dithionite-reduced carbon monoxide difference spectra of CaCYP51 proteins (panel D).

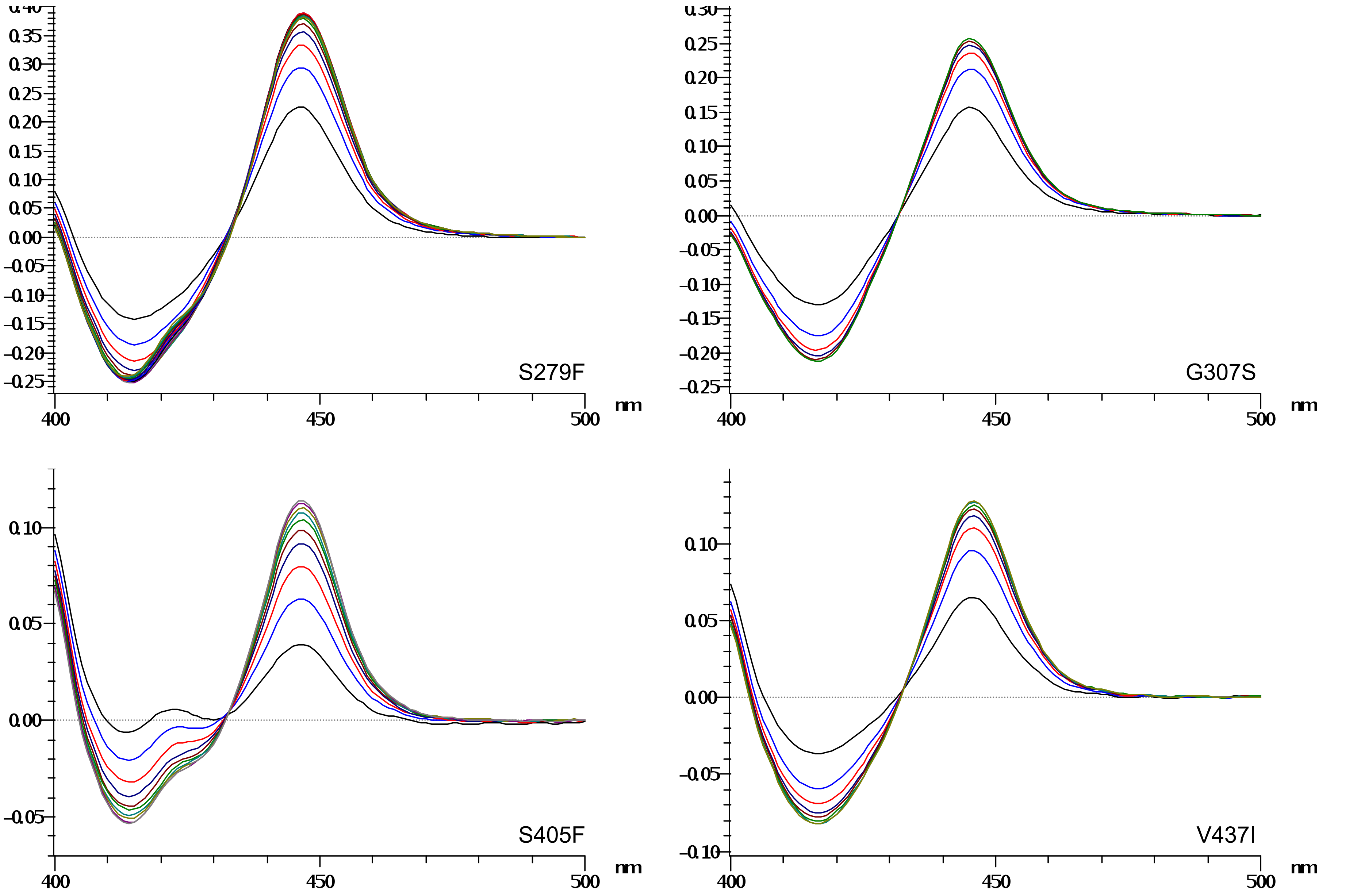

Figure S2 Dithionite-reduced carbon monoxide difference spectra of CaCYP51 proteins (panel E).

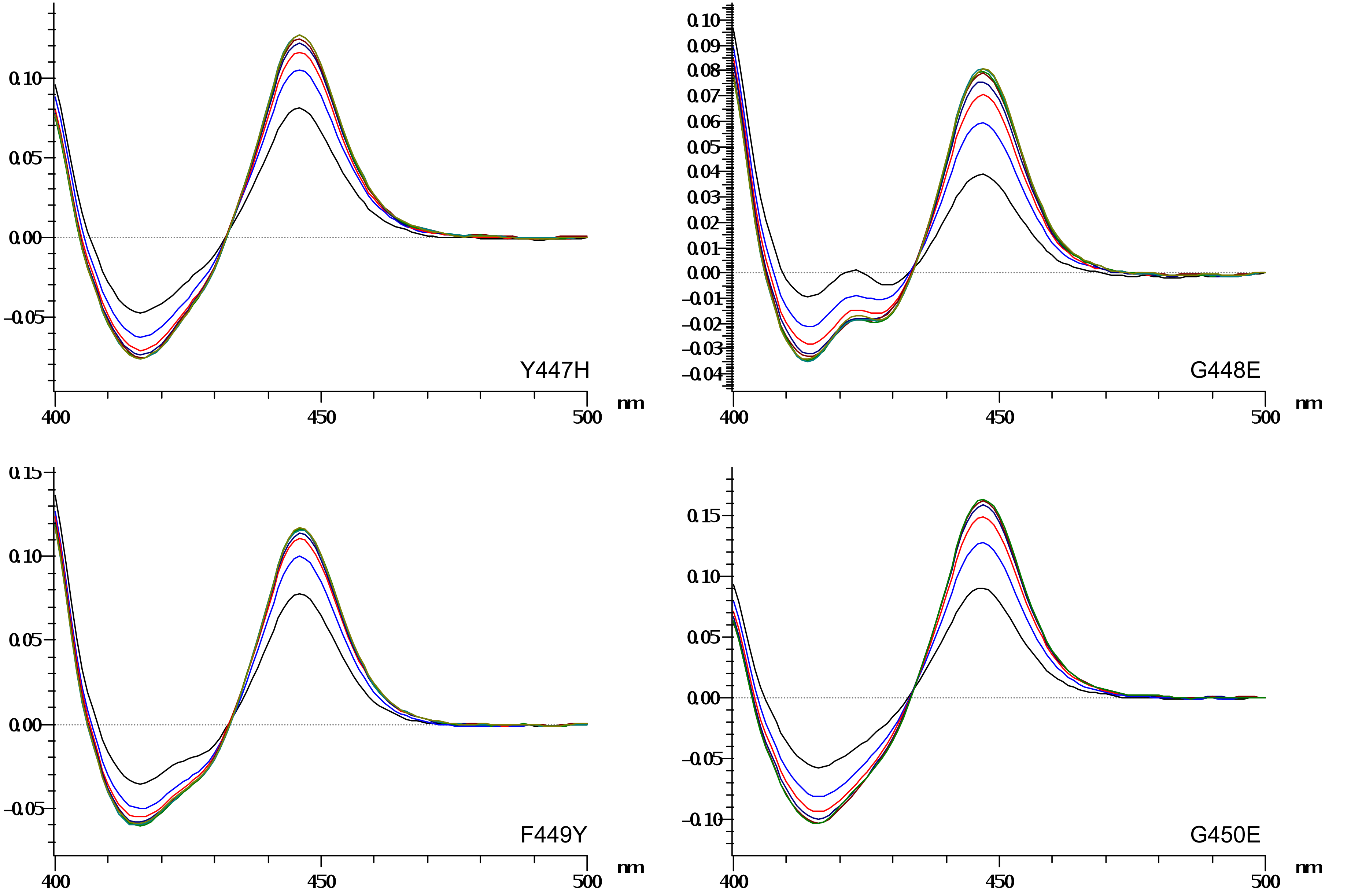

Figure S2 Dithionite-reduced carbon monoxide difference spectra of CaCYP51 proteins (panel F).

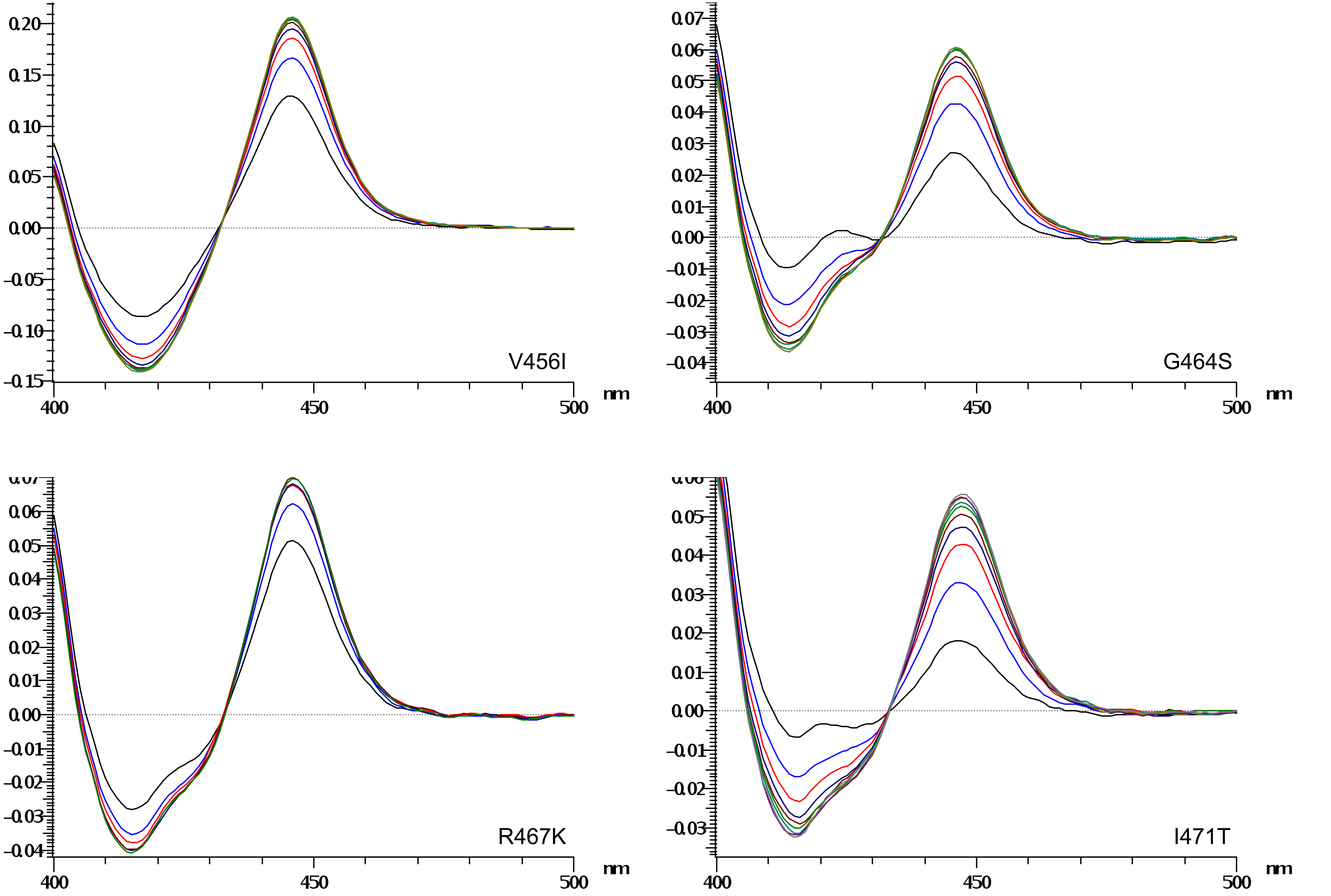

Figure S2 Dithionite-reduced carbon monoxide difference spectra of CaCYP51 proteins (panel G).

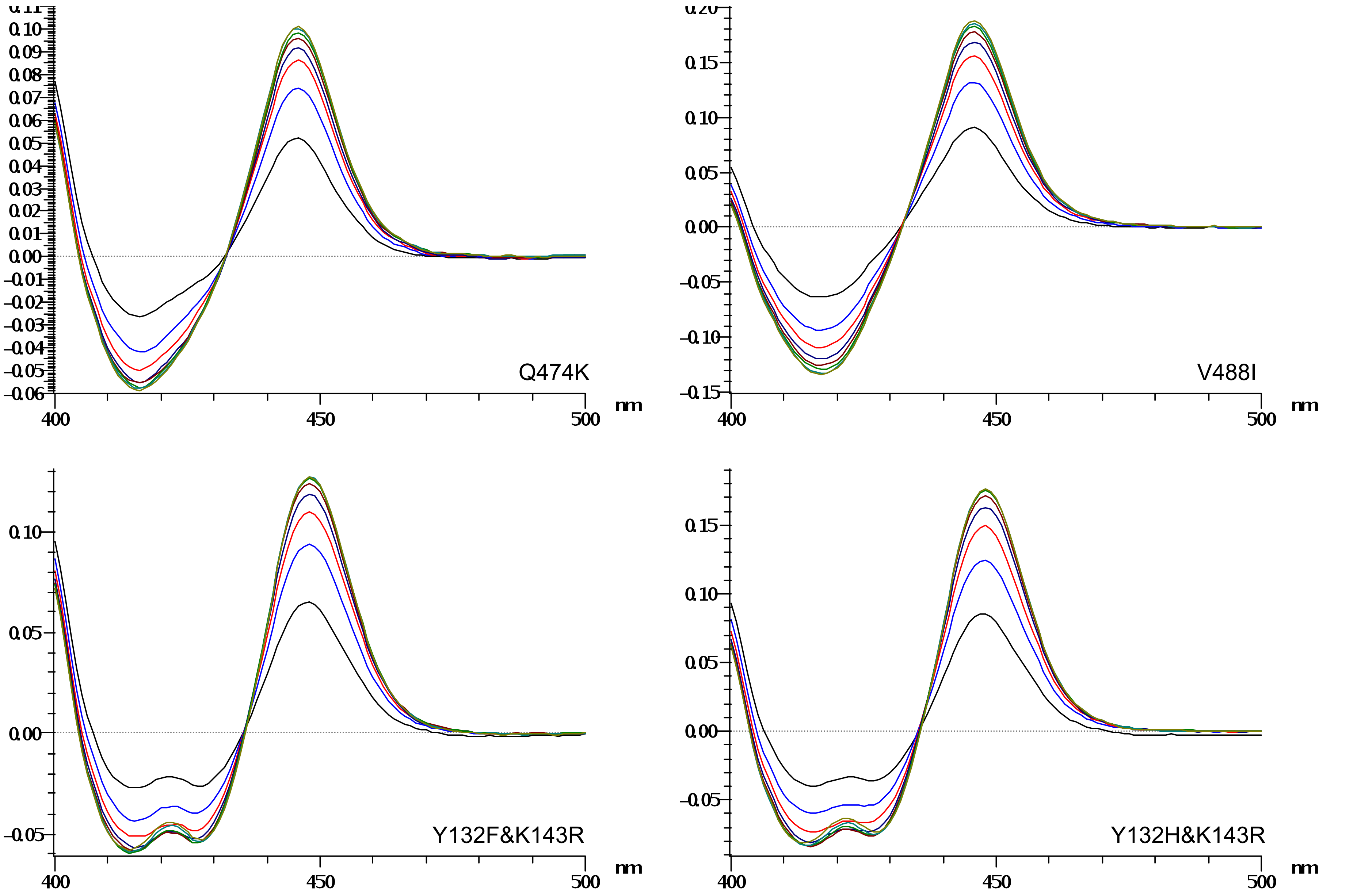

Figure S2 Dithionite-reduced carbon monoxide difference spectra of CaCYP51 proteins (panel H).

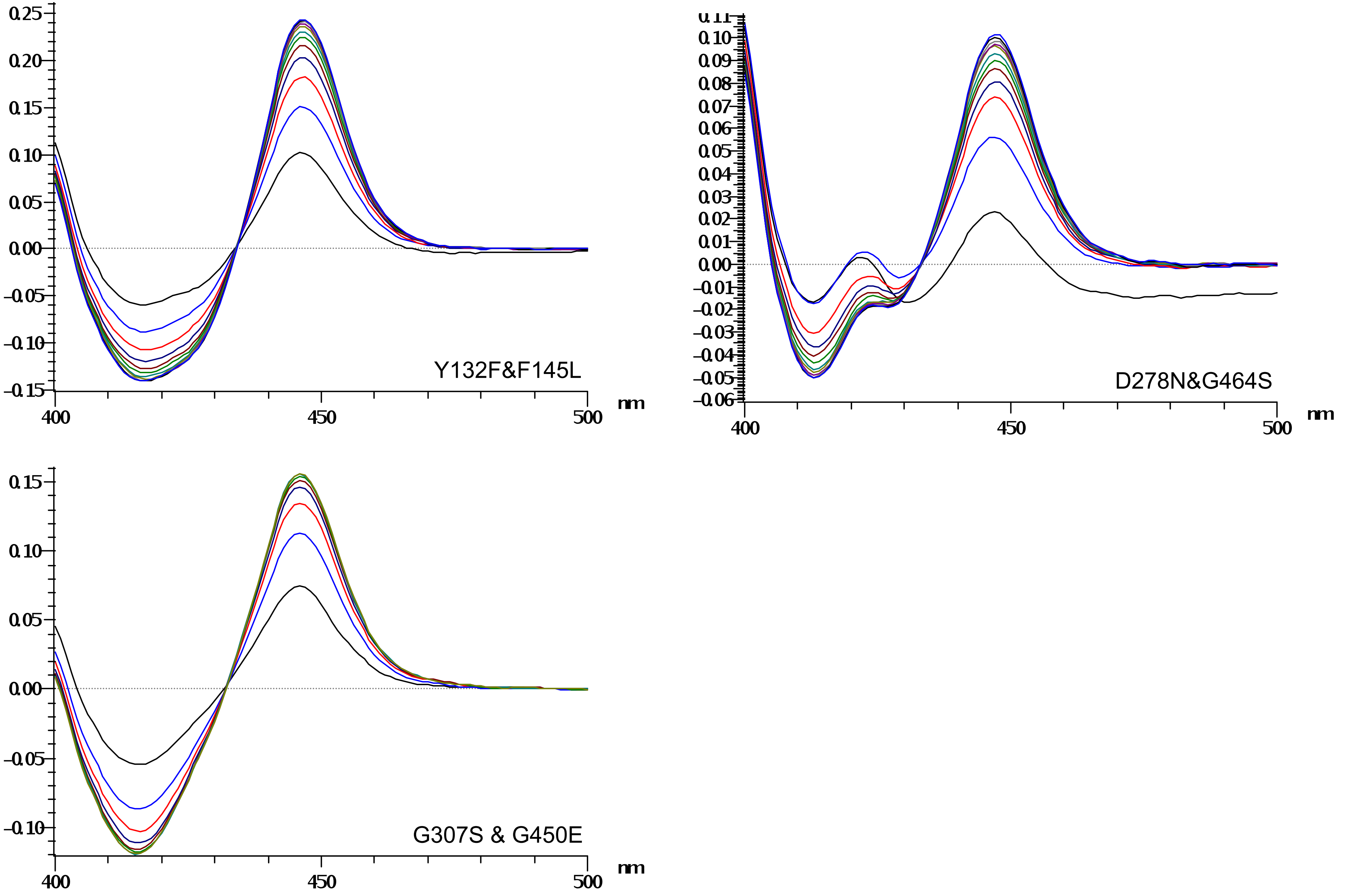

Figure S3 Fluconazole binding difference spectra with 4 μM CaCYP51 proteins (panel A).

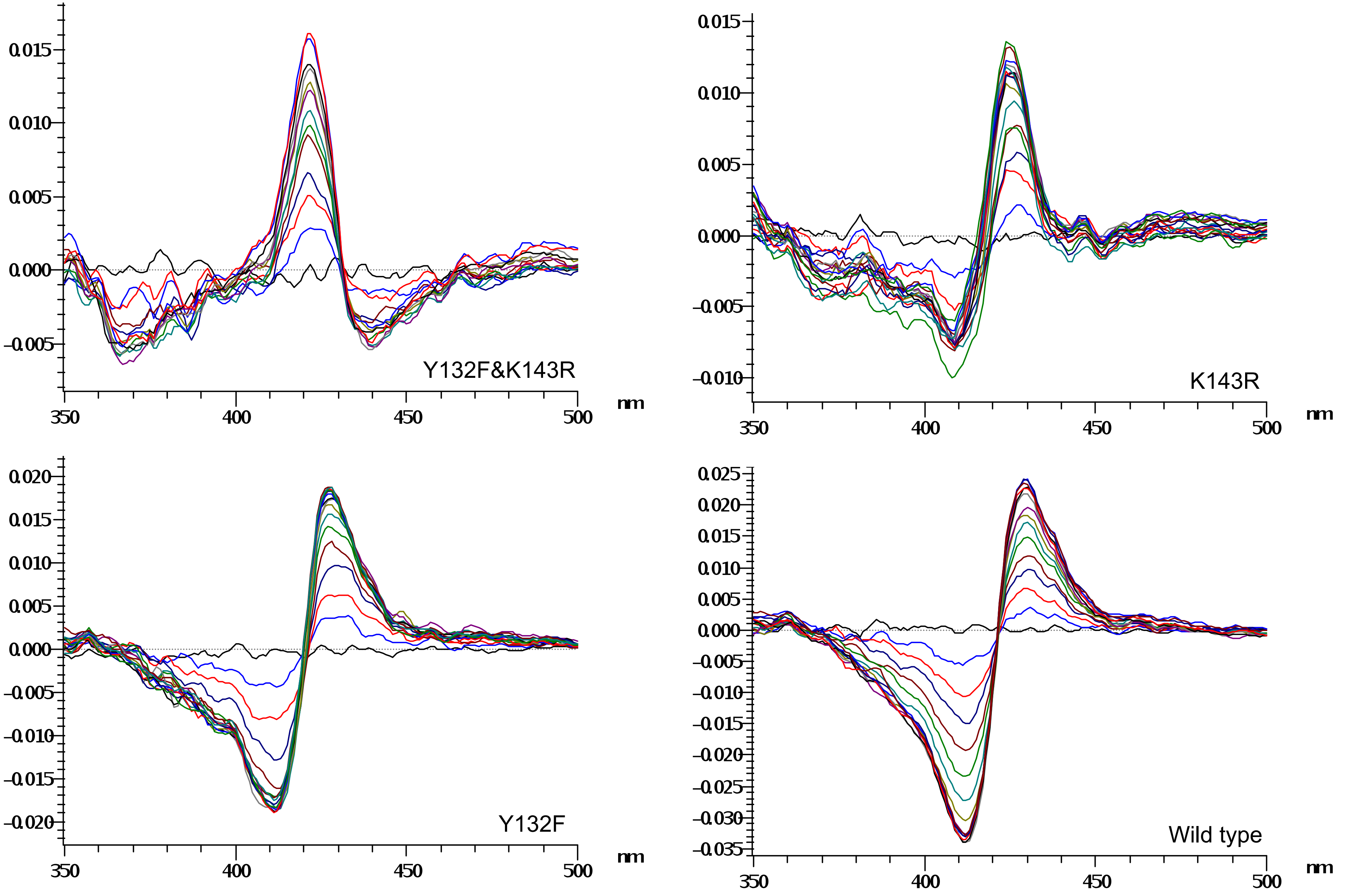

Figure S3 Fluconazole binding difference spectra with 4 μM CaCYP51 proteins (panel B).

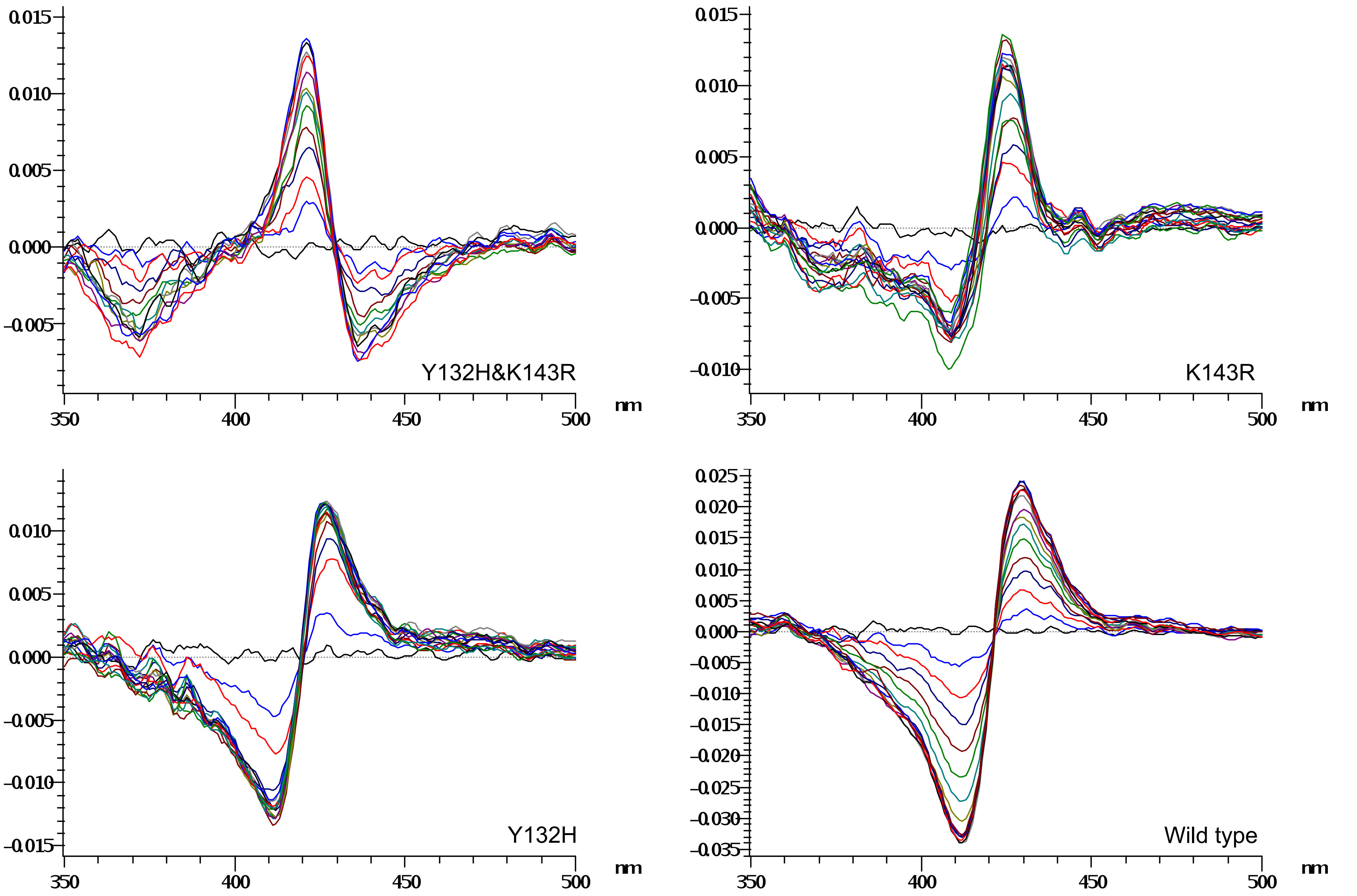

Figure S3 Fluconazole binding difference spectra with 4 μM CaCYP51 proteins (panel C).

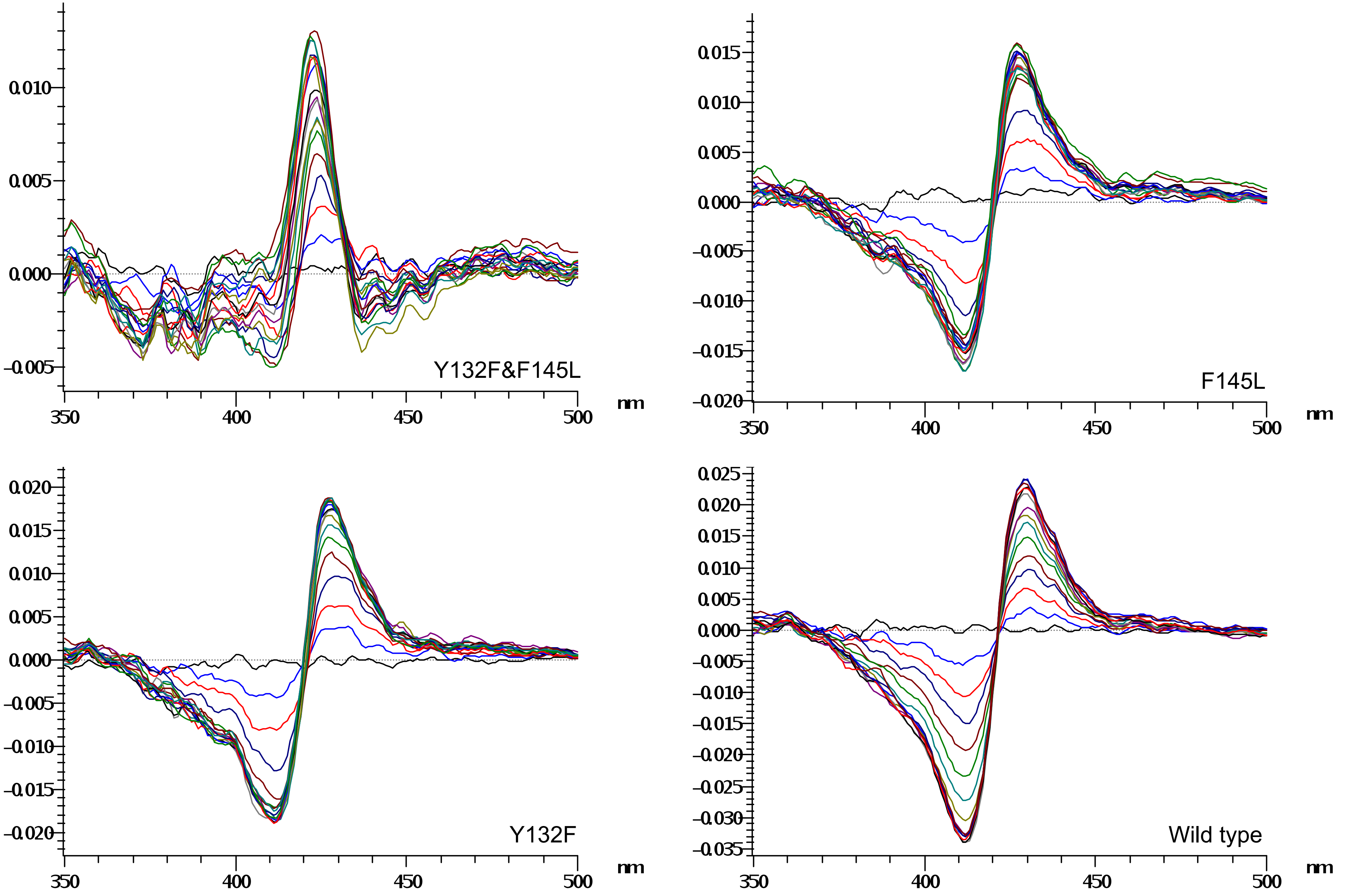

Figure S4 Residual CaCYP51 activity in the presence of 4 μM azole antifungal agent (panel A).

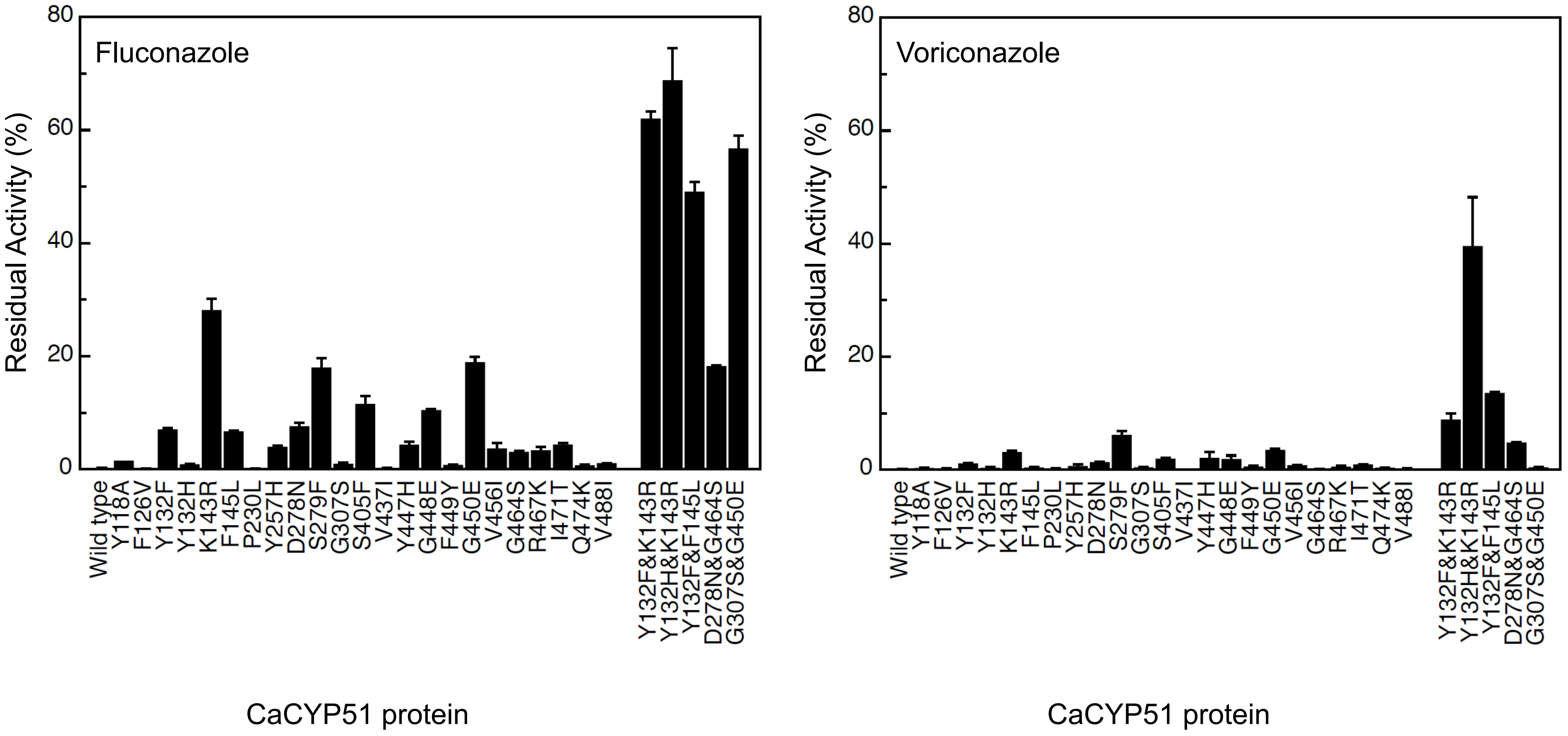

Figure S4 Residual CaCYP51 activity in the presence of 4 μM azole antifungal agent (panel B).

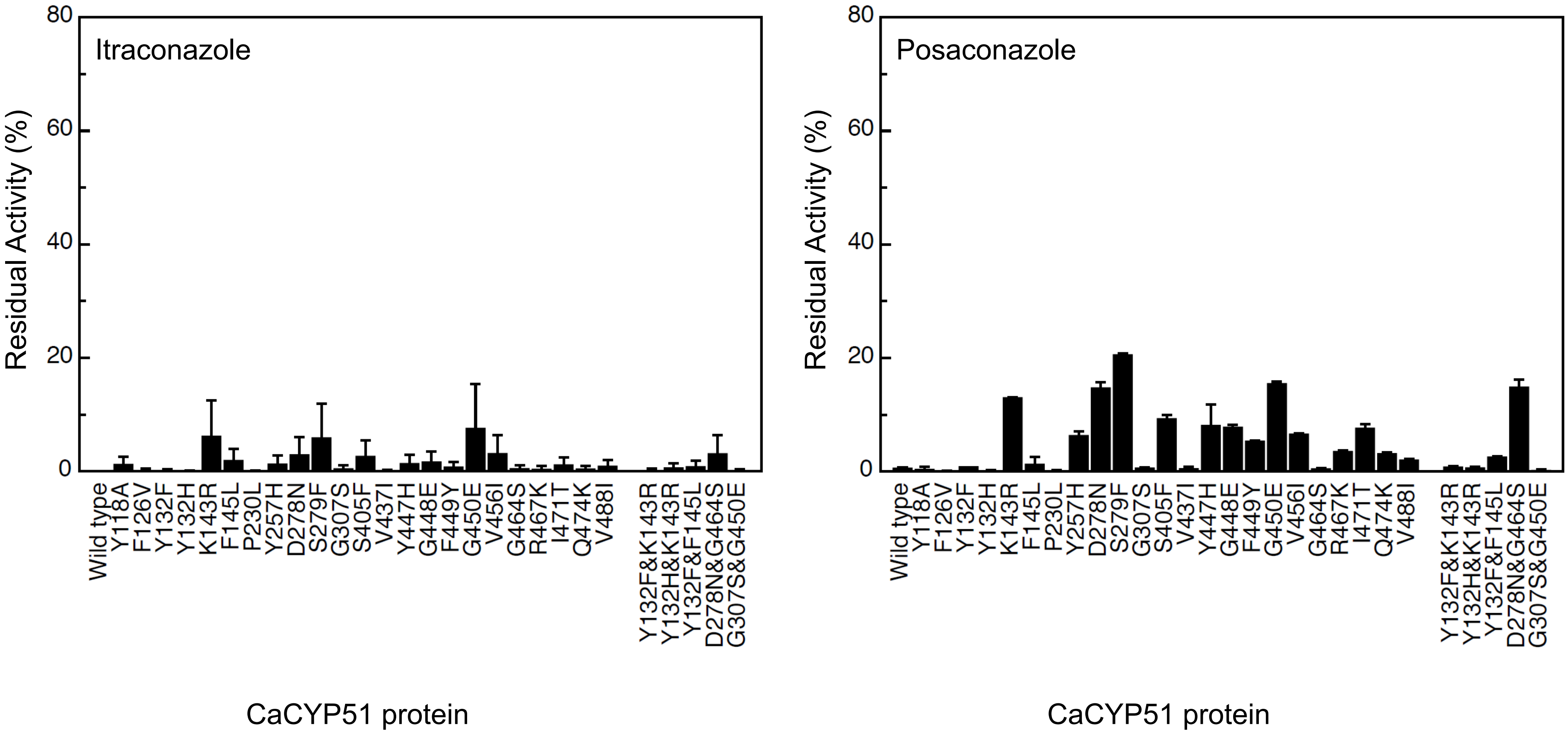

Figure S5 Chemical structures of azole antifungals.

Fluconazole
[MW 306]

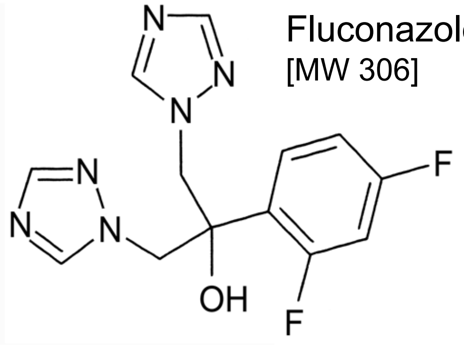

Voriconazole
[MW 349]

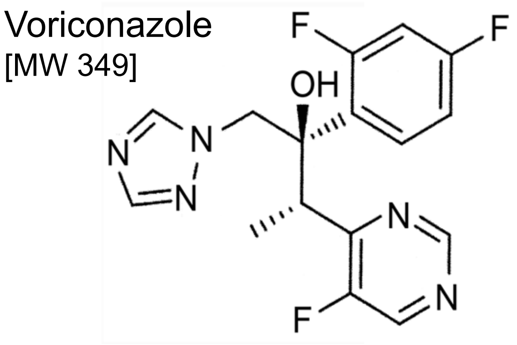

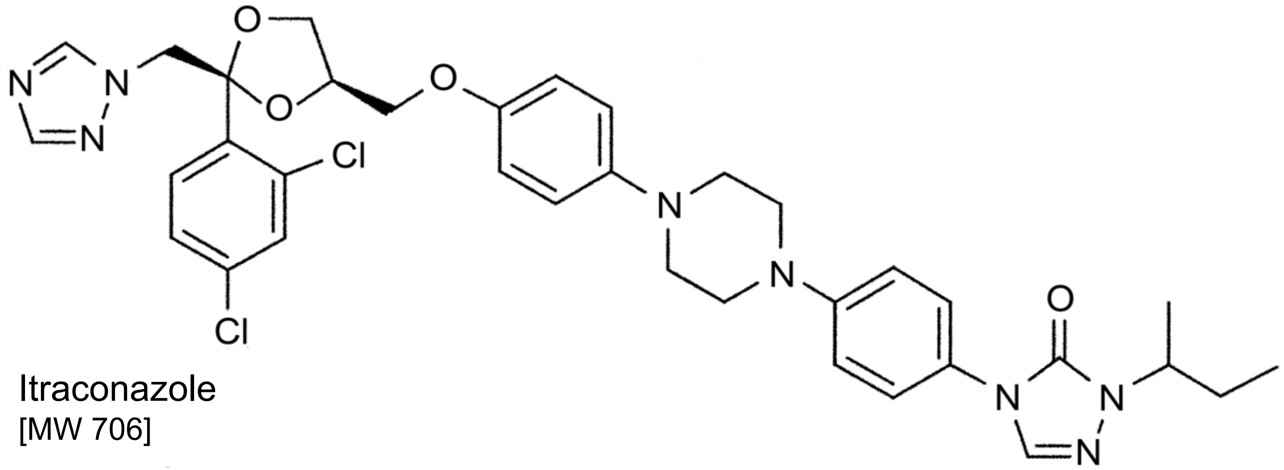

Itraconazole
[MW 706]

Posaconazole
[MW 701]

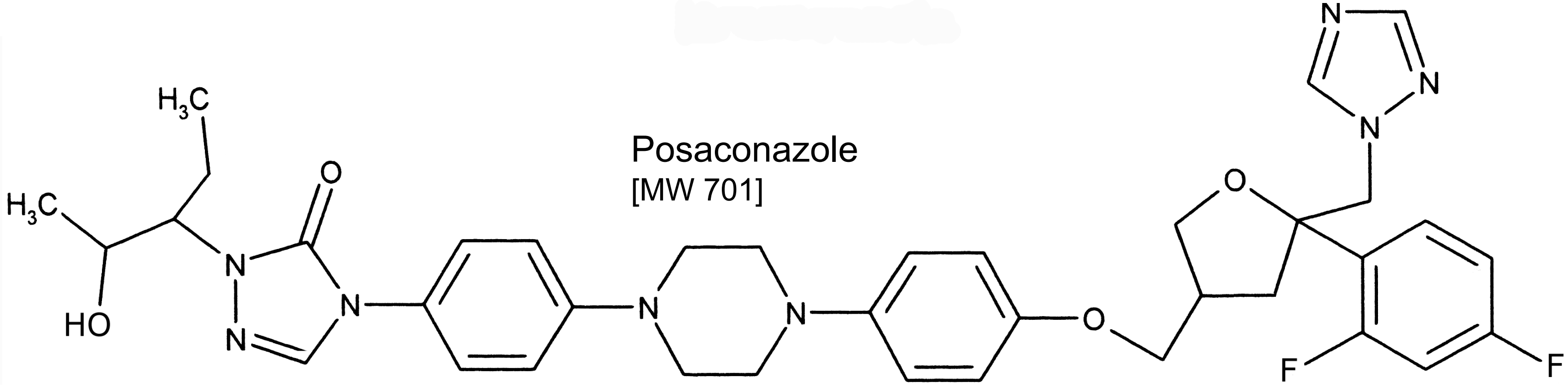

Supplement: Supplemental file 1 [file AAC.02586-18-s0001.pdf]
